# Supplementary material for: Bile Acid Detergency as Determinant of Liver Pathology in a Humanized Mouse Model of Progressive Familial Intrahepatic Cholestasis Type 3
Source: Cell Mol Gastroenterol Hepatol. 2026 Apr 8;20(8):101783. doi: 10.1016/j.jcmgh.2026.101783 (PMC13194600; doi:10.1016/j.jcmgh.2026.101783)
Supplement: Supplementary Material [file mmc1.pdf]

SUPPLEMENTAL MATERIAL

Supplemental Table S1. Primary probe sequences used in RNA fluorescence in situ hybridization (FISH) assay.

Gene	Probe_ID	Sequence 5'-3' with p27 3' initiator sequence (tttCATCATCAT)
m*S100a4*	1	AACCTTTTATTGAATTTGCTCAGCACTGTGCACATGTGCtttCATCATCAT
	2	AGTAAGGCACTATGCTCACAGCCAACAGGGAAGATCCtttCATCATCAT
	3	TGGCAAACTACACCCCAACACTTCATCTGAGGAGTCTtttCATCATCAT
	4	CCTTATCTGGGCAGCCCTCAAAGAATTCATTGCACATtttCATCATCAT
	5	CAATGCAGGACAGGAAGACACAGTACTCCTGGAAGTCtttCATCATCAT
	6	CATTGTCCCTGTTGCTGTCCAAGTTGCTCATCACCTTtttCATCATCAT
	7	CCCTGGTCAGTAGCTCCTTGAGCTCTGTCTTGTTCAGtttCATCATCAT
	8	ACTTGTCACCCTCTTTGCCTGAGTATTTGTGGAAGGTtttCATCATCAT
	9	TTGCCATGGTAACCGTTGAGACCAGACCAAGAGAGAGtttCATCATCAT

Supplemental Table S2. Taqman and SYBR Green PCR primer and probe sequences.

Gene		Taqmanoligo's (5'→3')
Col1a1	Fw	CGG CTC CTG CTC CTC TTA GG
	Rv	CTG ACT TCA GGG ATG TCT TCT TGG
	Probe	CCA CTG CCC TCC TGA CGC ATG G
Mmp12	Fw	ATC TTA GAG CAG TGC CCC AG
	Rv	CAT CCT CAC GCT TCA TGT CC
	Probe	TCA AGA TGG ATG AAG CGG TAC CTC AC
Timp1	Fw	TCT GAG CCC TGC TCA GCA A
	Rv	AAC AGG GAA ACA CTG TGC ACA C
	Probe	CCA CAG CCA GCA CTA TAG GTC TTT GAG AAA GC
Tnf	Fw	GTA GCC CAC GTC GTA GCA AAC
	Rv	AGT TGG TTG TCT TTG AGA TCC ATG
	Probe	CGC TGG CTC AGC CAC TCC AGC
Krt19	Fw	CAC CTA CCT TGC TCG GAT TGA
	Rv	CCG TGA CTT CGG TCT TGC TT
	Probe	ACA CCC AGG TCG CCG TCC ACT
Ppig	Fw	CAG ATC GAG GGA TCG ATT CAG
	Rv	TCA CCA CTT GAC ACC CTC ATT C
	Probe	CTC CTC CAC ATT GGA GAC AAG AGA TGC A

Gene		SYBR Green oligo's (5'→3')
Pemt	Fw	AGG AGT CCA GAG TGA CCA CAT TTC
	Rv	AGG AGA GCA ACC ACG TAG AC
Pcyt1a	Fw	CTG CAG TGA TGA GCT AAC GC
	Rv	GGC GAC AAA ATC AAT CCG GTG
Ppig	Fw	GGG GAT AAA GGT CCA GCG T
	Rv	AGA CAA CTC TTC CAG CAG GT
Pla2g4a	Fw	CAG CAC ATT ATA GTG GAA CAC CA
	Rv	AGT GTC CAG CAT ATC GCC AAA
Lpcat3	Fw	GGC CTC TCA ATT GCT TAT TT
	Rv	AGG ATG AGG AAC TGA AGA AC
