# Supplementary material for: Bile Acid Detergency as Determinant of Liver Pathology in a Humanized Mouse Model of Progressive Familial Intrahepatic Cholestasis Type 3
Source: Cell Mol Gastroenterol Hepatol. 2026 Apr 8;20(8):101783. doi: 10.1016/j.jcmgh.2026.101783 (PMC13194600; doi:10.1016/j.jcmgh.2026.101783)
Supplement: Extended PDF [file mmc2.pdf]

cmgh
CELLULAR AND MOLECULAR GASTROENTEROLOGY AND HEPATOLOGY

ORIGINAL RESEARCH

Bile Acid Detergency as Determinant of Liver Pathology in a Humanized Mouse Model of Progressive Familial Intrahepatic Cholestasis Type 3

Lixin Ke,[1] Niels L. Mulder,[1] Milaine V. Hovingh,[1] Rick Havinga,[1] Ellen Weersing,[2] Hilde D. de Vries,[1] Krisztina de Bruyn,[3] Kirill Ustyantsev,[2] Eugene Berezikov,[2] Vincent W. Bloks,[1] Brecht Attema,[2] Anna Worthmann,[4] Joerg Heeren,[4] Justina C. Wolters,[1] Rachel Tiessen-Thomas,[5] Henkjan J. Verkade,[1] Folkert Kuipers,[2,3] and Jan Freark de Boer[1,3]

[1]Department of Pediatrics, University of Groningen, University Medical Center Groningen, Groningen, The Netherlands; [2]European Research Institute for the Biology of Aging (ERIBA), University of Groningen, University Medical Center Groningen, Groningen, The Netherlands; [3]Department of Laboratory Medicine, University of Groningen, University Medical Center Groningen, Groningen, The Netherlands; [4]Department of Biochemistry and Molecular Cell Biology, University Medical Center Hamburg-Eppendorf, Hamburg, Germany; and [5]Royal GD, Deventer, The Netherlands

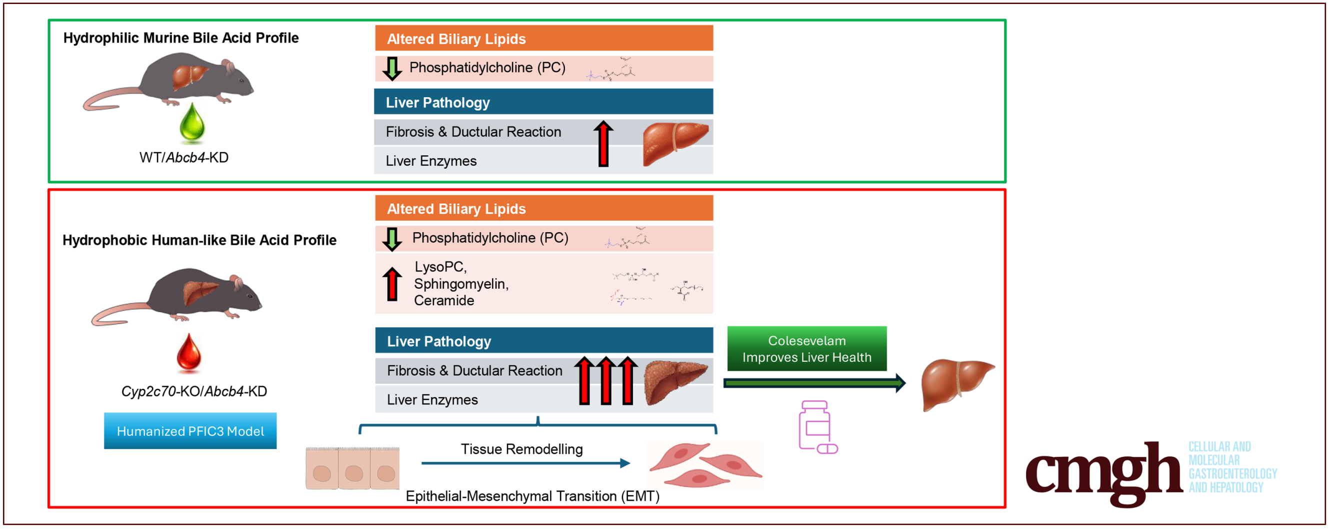

SUMMARY

A new mouse model for progressive familial intrahepatic cholestasis type 3 has been generated by *Abcb4* knockdown in *Cyp2c70*-knockout mice with a human-like bile acid composition, revealing aggravated liver injury that resembles the human disease and demonstrating the therapeutic benefit of bile acid sequestration.

WHAT YOU NEED TO KNOW

Background: Progressive familial intrahepatic cholestasis type 3 (PFIC3), caused by impaired activity of ABCB4 responsible for hepatobiliary transport of phosphatidylcholines (PC), is a severe progressive liver disease with limited treatment options, in part due to the absence of adequate animal models. This study aimed to develop and characterize a mouse model (*Cyp2c70*-KO/*Abcb4*-KD) that reflects PFIC3 pathology observed in humans.

Impact: The human-like bile acid composition in *Cyp2c70*-KO/*Abcb4*-KD mice profoundly aggravates liver pathology, conceivably due to the augmented extrusion of non-PC phospholipids by hydrophobic bile acids from membranes into bile.

Future Directions: The humanized PFIC3 model is anticipated to accelerate the development of novel therapies for PFIC3.

BACKGROUND & AIMS: Progressive familial intrahepatic cholestasis type 3 (PFIC3) is caused by impaired activity of ABCB4 that transports phosphatidylcholines (PC) from hepatocytes into bile. *Abcb4*-knockout (KO) mice have been generated, but hydrophilic muricholic acids in their bile acid (BA) pool may limit pathology and thereby hamper translation to human PFIC3. Therefore, we addressed whether *Abcb4* knockdown (*Abcb4*-KD) in the livers of *Cyp2c70*-KO mice with a human-like BA composition leads to liver pathology that more closely resembles human PFIC3.

METHODS: Expression of *Abcb4* gene was suppressed in livers of *Cyp2c70*-KO/L-Cas9tg mice and control-Cas9tg mice using CRISPR/Cas9-technology to assess short- and long-term consequences. The BA sequestrant colesevelam was mixed into the chow diet (2% w/w) of *Cyp2c70*-KO mice with or without *Abcb4*-KD, and the effects were evaluated after 6 weeks.

RESULTS: *Abcb4*-KD strongly reduced biliary phospholipid:BA ratios in both *Cyp2c70*-KO and control mice. However, plasma transaminases elevations, liver fibrosis, and ductular reactions were markedly aggravated by *Abcb4*-KD in the context of a human-like BA composition. Importantly, the biliary lipid compositions differed markedly between the 2 *Abcb4*-KD models. Reduced biliary PC and increased concentrations of lysophosphatidylcholine, sphingomyelin, and ceramide were associated with the severity of liver disease only in *Cyp2c70*-KO/*Abcb4*-KD mice. Gene set enrichment analysis indicated that epithelial-mesenchymal transition was induced in the livers of *Cyp2c70*-KO/*Abcb4*-KD mice compared with control *Abcb4*-KD mice. BA sequestration reduced biliary BA secretion rates and mitigated liver pathology in *Cyp2c70*-KO/*Abcb4*-KD mice.

CONCLUSIONS: The presence of a human-like BA composition profoundly aggravates liver pathology upon *Abcb4* reduction in mice, conceivably due to the augmented extrusion of non-PC phospholipids by hydrophobic BAs from membranes into bile. This humanized PFIC3 model is anticipated to accelerate the development of novel therapies for PFIC3 and potentially other cholestatic liver diseases. *(Cell Mol Gastroenterol Hepatol 2026;20:101783; https://doi.org/10.1016/j.jcmgh.2026.101783)*

Keywords: *Abcb4*; Bile Acid Hydrophobicity; PFIC3; Translational Murine Model.

Progressive familial intrahepatic cholestasis 3 (PFIC3) is a genetic liver disease characterized by persistent and often severe cholestasis. PFIC3 symptoms can become apparent from 1 month to over 20 years of age (mean age, around 3.5 years).[1] About 50% of patients develop liver cirrhosis, portal hypertension, and end-stage liver disease, requiring liver transplantation.[2] Loss-of-function variants in the *ABCB4* gene, which encodes the phosphatidylcholine (PC) transporter ABCB4 (MDR3 in humans; MDR2 in mice), underlie PFIC3 (also referred to as MDR3-deficiency).[3,4] ABCB4 is localized at the canalicular membrane of hepatocytes, where it functions as a floppase that translocates PCs from the inner to the outer leaflet, thereby facilitating their secretion into the bile[5] to allow the formation of mixed micelles with bile acids (BAs) and cholesterol. The mixed micelles protect the epithelial cells lining the biliary tree from the detergent activity of BAs.[6,7]

Currently, treatment options for patients with PFIC3 are limited. Ursodeoxycholic acid (UDCA) improves liver function and clinical symptoms to some extent in about 50% of patients with PFIC3.[8] The efficacy of UDCA appears to depend on the type of mutation: patients with a missense mutation allowing residual ABCB4 activity show a better response than those with a complete loss of ABCB4 function.[9,10] In part of the patients on long-term UDCA treatment, disease progression is complicated by cirrhosis, portal hypertension, and liver failure.[11] Ileal bile acid transporter (IBAT) inhibitors have recently received European Medicines Agency (EMA) approval for the treatment of PFIC and United States Food and Drug Administration (FDA) approval for the treatment of cholestatic pruritus in patients with PFIC.[12,13] However, whether IBAT inhibitors actually delay development of liver damage and prevent or postpone the need for liver transplantation in patients with PFIC3 is still unknown. In addition, gene therapy may represent a promising therapeutic strategy for PFIC3,[14–16] but its potential requires comprehensive preclinical and clinical evaluation. Currently, liver transplantation is still the only curative treatment option for the majority of patients,[17,18] underscoring the unmet need for alternative treatment strategies for patients with PFIC3.

Abcb4-knockout (KO) mice are genetic murine equivalents of human PFIC3.[19] However, although both *Abcb4*-KO mice and patients with PFIC3 display impaired biliary PC secretion, disease progression appears to be much slower in the mouse model, and symptoms are milder than those seen in patients with PFIC3.[20–22] Actually, liver pathology that develops in *Abcb4*-KO mice resembles the features of primary sclerosing cholangitis (PSC) more than those of PFIC3 and, therefore, *Abcb4*-KO mice have been advocated to represent a model for PSC.[19] This divergence between murine and human PFIC3 is probably related to the fact that the murine BA pool is much less cytotoxic than that of humans. The murine BA pool consists of 40% to 60% of

Abbreviations used in this paper: AAV, adeno-associated virus; ALP, alkaline phosphatase; ALT, alanine aminotransferase; AST, aspartate aminotransferase; BA, bile acid; CA, cholic acid; CDCA, chenodeoxycholic acid; CK19, cytokeratin-19; cPLA2, cytosolic phospholipase A2; DAB, 3,3'-diaminobenzidine; DAPI, 4′,6-diamidino-2-phenylindole; DCA, deoxycholic acid; DMS, differential mobility spectrometry; EMA, European Medicines Agency; EMT, epithelial mesenchymal transition; FDA, United States Food and Drug Administration; FIA-MS/MS, flow injection analysis-tandem mass spectrometry; FISH, fluorescent in situ hybridization; FFPE, formalin-fixed paraffin-embedded; GSEA, gene set enrichment analysis; H&E, hematoxylin and eosin; HET, heterozygous; IBAT, ileal bile acid transporter; KD, knockdown; KEGG, Kyoto Encyclopedia of Genes and Genomes; KO, knockout; LPC, lysophosphatidylcholine; MAPKs, mitogen-activated protein kinases; MCAs, muricholic acids; MUP, major urinary protein; PBS, phosphate-buffered saline; PCA, principal component analysis; PC, phosphatidylcholine; PE, phosphatidylethanolamines; PEI, polyethylenimine; PFIC3, progressive familial intrahepatic cholestasis type 3; PL, phospholipid; PLS-DA, partial least squares discriminant analysis; PSC, primary sclerosing cholangitis; RNA-seq, RNA sequencing; RT-qPCR, quantitative real-time polymerase chain reaction; scAAV, self-complementary adeno-associated virus; sgRNA, single-guide RNA; SM, sphingomyelin; SRFG, Sirius Red and Fast Green; SSCTw, saline-sodium citrate containing Tween-20; TG, triglyceride; TGF-β, transforming growth factor-β; THBAs, tetrahydroxylated bile acids; TSA, tyramide signal amplification; UDCA, ursodeoxycholic acid; UHPLC-MS/MS, ultra-high performance liquid chromatography-tandem mass spectrometry; WT, wild type.

Most current article

© 2026 The Author(s). Published by Elsevier Inc. on behalf of American Gastroenterological Association Institute. This is an open access article under the CC BY license (http://creativecommons.org/licenses/by/4.0/).
2352-345X
https://doi.org/10.1016/j.jcmgh.2026.101783

hydrophilic, non-toxic, muricholic acids (MCAs), whereas cholic, deoxycholic, and chenodeoxycholic acids (CA, DCA, and CDCA, respectively) are the prominent BA species present in humans.[23] These relatively hydrophobic BAs have cytotoxic properties at high concentrations and may damage the cells lining the biliary tree, particularly when BA micelles are undersaturated with phospholipids. Thus, physiologically very relevant differences in BA metabolism exist between mice and humans. CYP2C70 has recently been identified as the enzyme responsible for synthesizing MCAs in mice,[23,24] and *Cyp2c70*-KO mice, having a more hydrophobic BA profile that much closer mimics that of humans, have been generated.[25,26] These 'humanized' mice provide unique opportunities to evaluate the efficacy of potential therapeutic approaches for BA-related liver diseases, including PFIC3.

We aimed to develop and characterize a mouse model (*Cyp2c70*-KO/*Abcb4*-knockdown [KD]) that more accurately reflects the PFIC3 pathology observed in humans with *ABCB4*-deficiency. Furthermore, because recent studies have highlighted the therapeutic potential of modulating the enterohepatic circulation of BAs for the treatment of cholestatic liver diseases,[27–30] we also assessed the impact of BA sequestration on liver pathology in this new PFIC3 mouse model.

Results

Abcb4 KD Results in Altered Biliary Lipid Profile in Mice With a Human-Like BA Composition

To evaluate the early effects of *Abcb4* deficiency in the context of a human-like BA pool, we sacrificed *Cyp2c70*-wild type (WT)/heterozygous (HET) (control) and *Cyp2c70*-KO mice expressing Cas9 in their livers at 10 days after injection with adeno-associated virus (AAV) encoding single-guide RNAs (sgRNAs) designed to inactivate the *Abcb4* gene (AAV-sg*Abcb4*) or with a control virus (AAV-Empty) (Figure 1*A*). Hepatic ABCB4 protein levels were decreased by 83.2% and 77.1% in control/*Abcb4*-KD and *Cyp2c70*-KO/*Abcb4*-KD mice compared with those in control and *Cyp2c70*-KO mice, respectively (Figure 1*B*), confirming efficient inactivation of the *Abcb4* gene. Accordingly, although bile flow remained unaffected (Figure 1*C*), total biliary phospholipid (PL) concentrations and PL:BA ratios were strongly decreased upon *Abcb4*-KD in both settings (Figure 1*D*). Interestingly, the PL:BA ratio was higher in *Cyp2c70*-KO/*Abcb4*-KD than in control/*Abcb4*-KD mice (Figure 1*D*–*F*). As expected, all *Cyp2c70*-KO mice showed high abundances of CDCA in bile and plasma, whereas MCAs were absent, translating into more hydrophobic BA pools than the control groups (Figure 1*G*–*L*). *Abcb4*-KD led to reduced contributions of 12α-hydroxylated BAs in the bile of *Cyp2c70*-KO mice only (Figure 1*I* and *L*).

Mass spectrometry was performed to determine the effects of *Abcb4*-KD on biliary lipid composition under the conditions employed. Partial least squares discriminant analysis (PLS-DA) on the lipid profiles obtained (575 lipid species) revealed clear separation between *Cyp2c70*-KO, control, *Cyp2c70*-KO/*Abcb4*-KD, and control/*Abcb4*-KD mice (Figure 2*A*), indicating that the composition of the circulating BA pool per se strongly impacts biliary lipid profiles, effects that are accentuated under conditions of impaired ABCB4 activity. The major components of biliary PL known to be transported by ABCB4 (ie, PC) was significantly decreased in both *Abcb4*-KD mice, which verified the efficiency of *Abcb4*-KD (Figure 2*B*). Interestingly, there was also a clear separation between *Cyp2c70*-KO/*Abcb4*-KD and control/*Abcb4*-KD mice with respect to their biliary PC profiles (Figure 2*C*), revealing that *Abcb4* inactivation in the context of a human-like BA composition results in the presence of a different PC profile in bile. Figure 2*D* presents these differences, revealing relatively stronger overall decreases in "bile-specific" PC (PC 16:0_18:1, PC 16:0_18:2 and PC 16:0_20:4) upon *Abcb4*-KD in control mice than in *Cyp2c70*-KO mice, with relative increases of "bile-aspecific" species (lysoPC, sphingomyelin (SM), and ceramide) in the latter. The expression of key genes in hepatic PC synthesis (ie, *Pemt* and *Pcyt1a*) was somewhat lower in *Cyp2c70*-KO/*Abcb4*-KD than in control/*Abcb4*-KD mice (Figure 2*E* and *F*), but hepatic PC synthesis is known not to control biliary PC secretion in mice.[31] The expression of genes involved in the formation and remodeling of lysoPC (*Lpcat3* down, *Pla2g4a* up) (Figure 2*G* and *H*) indicated altered PC conversion in *Abcb4*-KD mice. Surprisingly, triacylglycerols (eg, TG 50:2-FA 16:1, TG 50:2-FA 18:1, and TG 52:2-FA 16:0) were found in bile, and their presence was decreased in *Cyp2c70*-KO/*Abcb4*-KD mice compared with the other 3 groups (Figure 2*D*). The presence of triglycerides (TGs) and apolipoprotein B in the bile of obese patients with gallstones has been reported previously[32] and was interpreted to indicate the secretion of TG-rich lipoproteins into the bile. Lower TG levels in the bile of *Cyp2c70*-KO/*Abcb4*-KD mice may thus reflect altered hepatic lipid metabolism.

Associations Between Changes in Biliary Lipid Composition and Parameters of Liver Disease are Apparent Only in Mice With a Human-Like Bile Acid Profile

The development of liver pathology upon *Abcb4* inactivation appeared markedly aggravated in the context of human-like BA composition. Body weights were slightly lower, whereas liver and spleen weights were substantially higher in *Cyp2c70*-KO/*Abcb4*-KD mice than in AAV-Empty injected controls and control/*Abcb4*-KD mice (Figure 3*A*–*D*), suggesting the presence of more liver damage and a higher inflammatory state in the first. Indeed, plasma transaminases were only modestly elevated upon *Abcb4*-KD in control mice, whereas strong increases in plasma alanine aminotransferase (ALT) and aspartate aminotransferase (AST) levels were observed when *Abcb4* was inactivated in *Cyp2c70*-KO mice (Figure 3*E* and *F*).

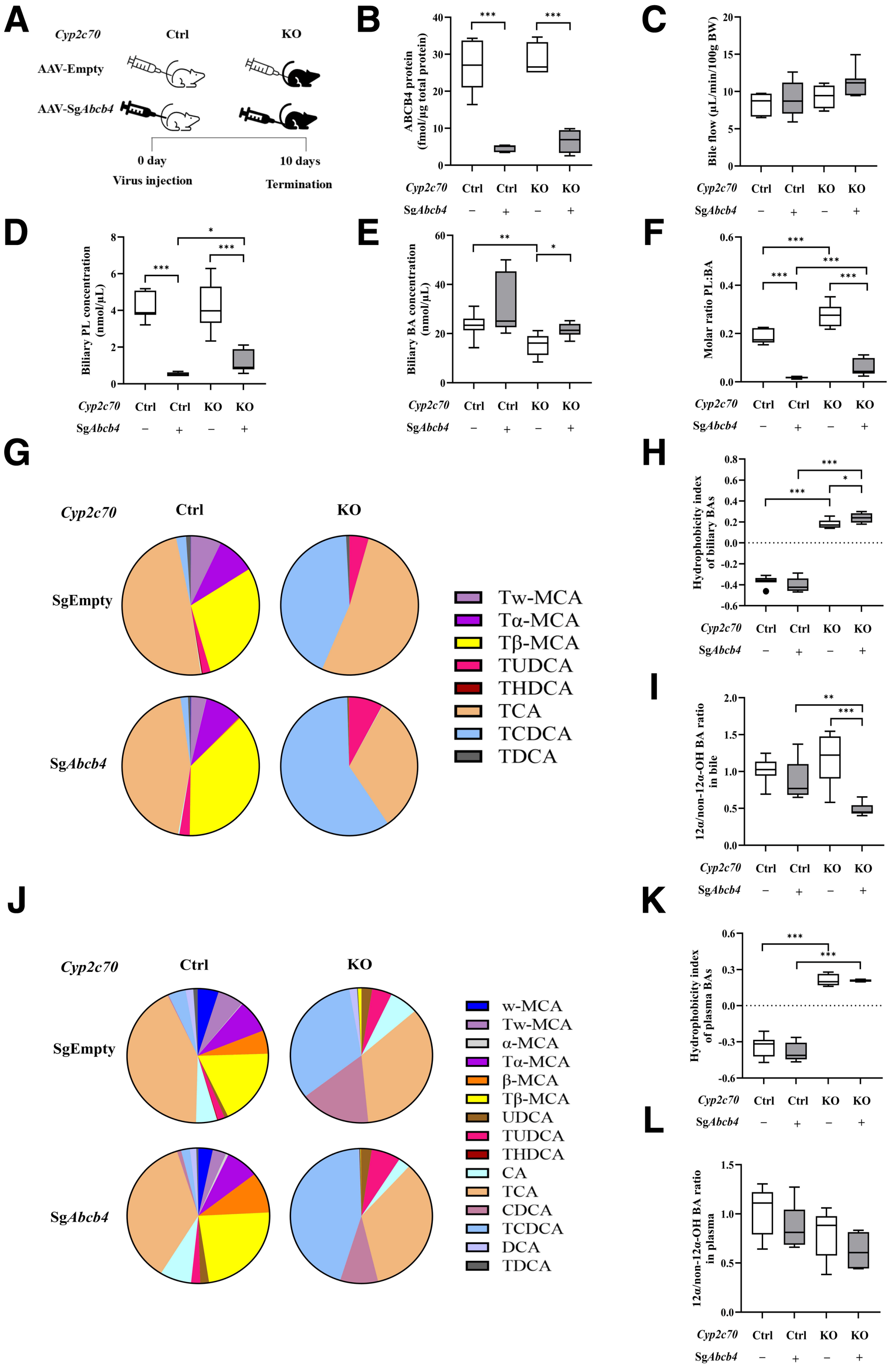

Figure 1. Ablation of hepatic *Abcb4* reduces biliary phospholipid secretion in male *Cyp2c70*-KO and control mice, and increases the hydrophobicity of biliary BAs in male *Cyp2c70*-KO mice at 10 days after virus injection. (*A*) Study design: male *Cyp2c70*-Ctrl/L-Cas9tg and *Cyp2c70*-KO/L-Cas9tg mice (n = 5–7 per group) were injected with AAV-Empty or AAV-sg*Abcb4* and sacrificed 10 days later. (*B*) Hepatic protein levels of ABCB4 determined using targeted proteomics. (*C*) Bile flow. (*D*) Total biliary PL concentration. (*E*) Total biliary BA concentration. (*F*) PL:BA molar ratios in bile. (*G*) Biliary BA profile. (*H*) Hydrophobicity index of biliary BAs. (*I*) Biliary 12α-/non12α-OH BA ratio. (*J*) Plasma BA profile. (*K*) Hydrophobicity index of plasma BAs. (*L*) Plasma 12α-/non12α-OH BA ratio. *$P < .05$, **$P < .01$, ***$P < .001$, according to Kruskal-Wallis H test followed by Conover post-hoc comparisons.

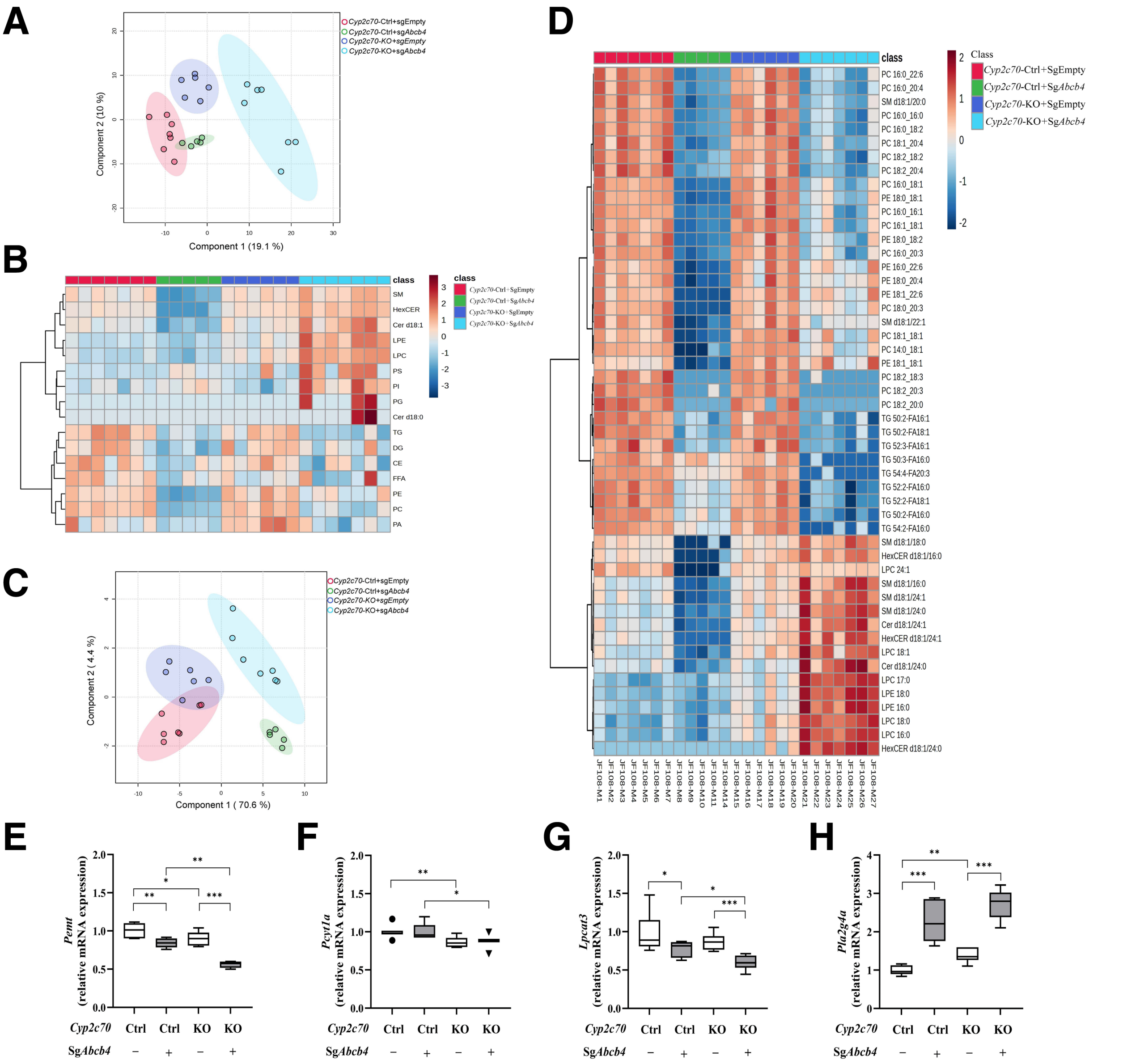

Figure 2. Ablation of hepatic *Abcb4* differentially modulates biliary lipid composition in male *Cyp2c70*-KO and control mice at 10 days after virus injection. (*A*) PLS-DA plot of biliary lipid species quantified by lipidomics. (*B*) Heatmap of the relative abundance of different lipids classes in bile as determined by lipidomics measurement. (*C*) PLS-DA plot of PC species present in bile quantified by lipidomics. (*D*) Heatmap of top 50 most significant differentially biliary lipids species (*red*, increased; *blue*, decreased). (*E–H*) Hepatic expression of genes involved in PC synthesis (*Pemt*, *Pcyt1a*) and metabolism towards LPC (*Lpcat3*, *Pla2g4a*). **P* < .05, ***P* < .01, ****P* < .001, according to Kruskal-Wallis H test followed by Conover post-hoc comparisons. CE, cholesterol esters; Cer, ceramide; DG, diacylglycerol; FFA, free fatty acids; HexCER, hexosylceramide; LPE, lysophosphatidylethanolamine; PA, phosphatidic acid; PE, phosphatidylethanolamine; PI, phosphatidylinositol; PG, phosphatidylglycerol; PS, phosphatidylserine; TG, triacylglycerol.

Moreover, plasma alkaline phosphatase (ALP) levels, a marker of cholangiocyte damage,[33] and plasma BA levels were not significantly impacted upon *Abcb4*-KD in control mice but were markedly elevated when *Abcb4* was inactivated in the context of a human-like BA pool composition (ie, in *Cyp2c70*-KO mice) (Figure 3*G* and *H*). In addition, hepatic expression of fibrogenic genes such as *Col1a1*, *Mmp12*, and *Timp1*, as well as expression of the cholangiocyte marker *Krt19* (encoding cytokeratin-19 [CK-19]), were much stronger induced in *Cyp2c70*-KO/*Abcb4*-KD mice than in control/*Abcb4*-KD mice, whereas the inflammatory cytokine *Tnf* was equally elevated upon *Abcb4*-KD in both genotypes (Figure 3*I*). Histological examination confirmed that single cell death, pigmented macrophages, and inflammation scores were higher, whereas collagen deposition and ductular reactions were more evident in the

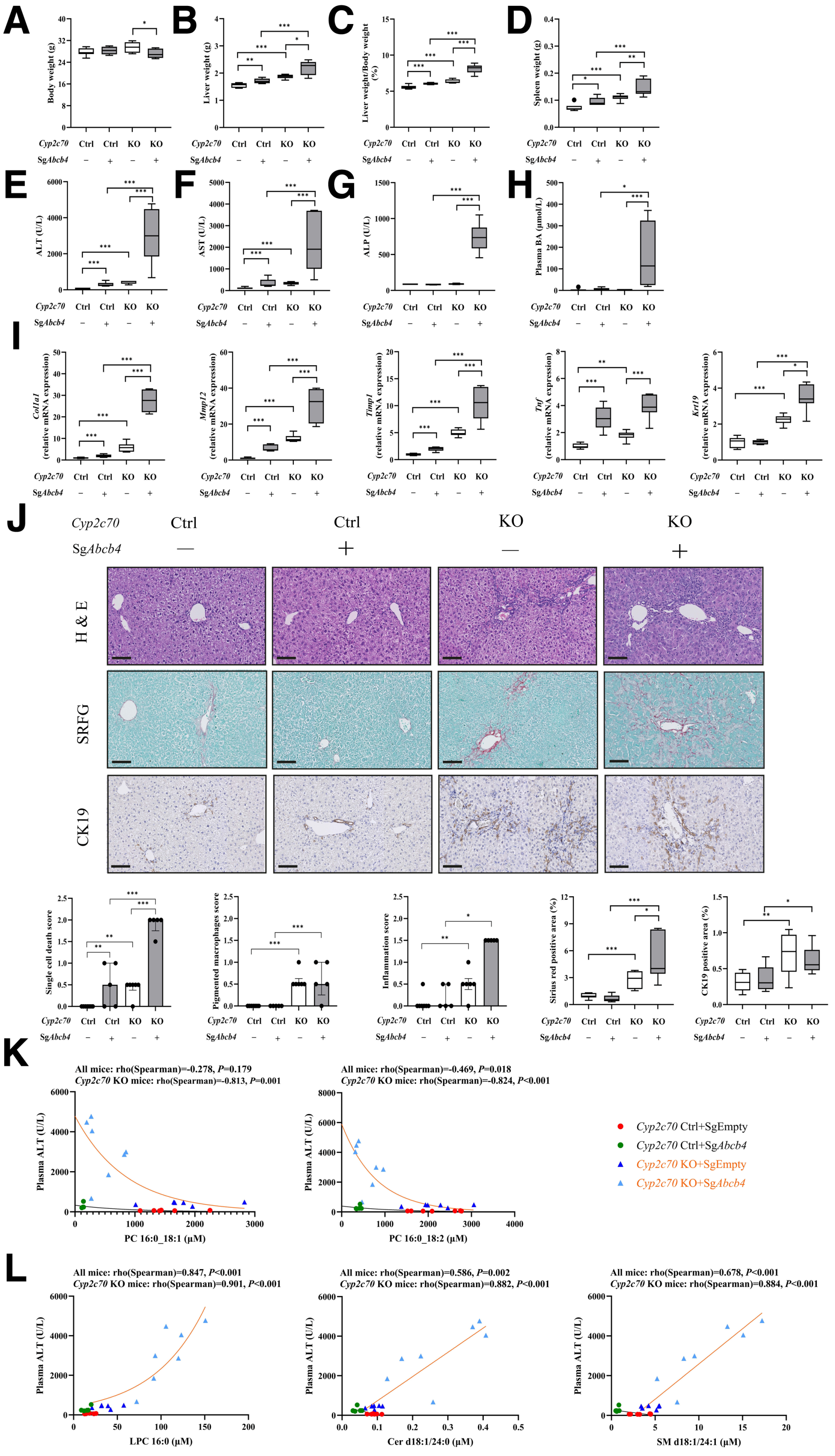

Figure 3. Reduction of hepatic *Abcb4* expression rapidly induces liver pathology in male *Cyp2c70*-KO mice at 10 days after virus injection. (*A*) Body weight. (*B*) Liver weight. (*C*) The ratio of liver weight to body weight. (*D*) Spleen weight. (*E–H*) Plasma levels of transaminases (ALT, AST), ALP, and BAs. (*I*) Hepatic mRNA expression of fibrogenic genes (*Col1a1*, *Mmp12*, *Timp1*), 1 pro-inflammatory gene (*Tnf*), and cholangiocyte marker (*Krt19*). (*J*) Representative images and quantification of liver sections stained with H&E, SRFG, and anti-CK19 antibody (bars represent 100 μm). Associations between biliary (*K*) PC, (*L*) LPC, ceramide, and SM with plasma ALT levels. *$P < .05$, **$P < .01$, ***$P < .001$, according to Kruskal-Wallis H test followed by Conover post-hoc comparisons. Cer, ceramide.

livers of *Cyp2c70*-KO/*Abcb4*-KD mice than in those of control/*Abcb4*-KD mice (Figure 3*J*).

To assess whether the specific changes in biliary lipid composition upon *Abcb4* suppression were related to varying degrees of liver disease, as reflected by interindividual variations in plasma ALT, AST, and ALP (see Figure 3*E–G*), we performed correlation analyses on all groups combined and on mice with *Cyp2c70*-KO background only. Interestingly, we found a clear negative relationship between biliary PC concentrations and

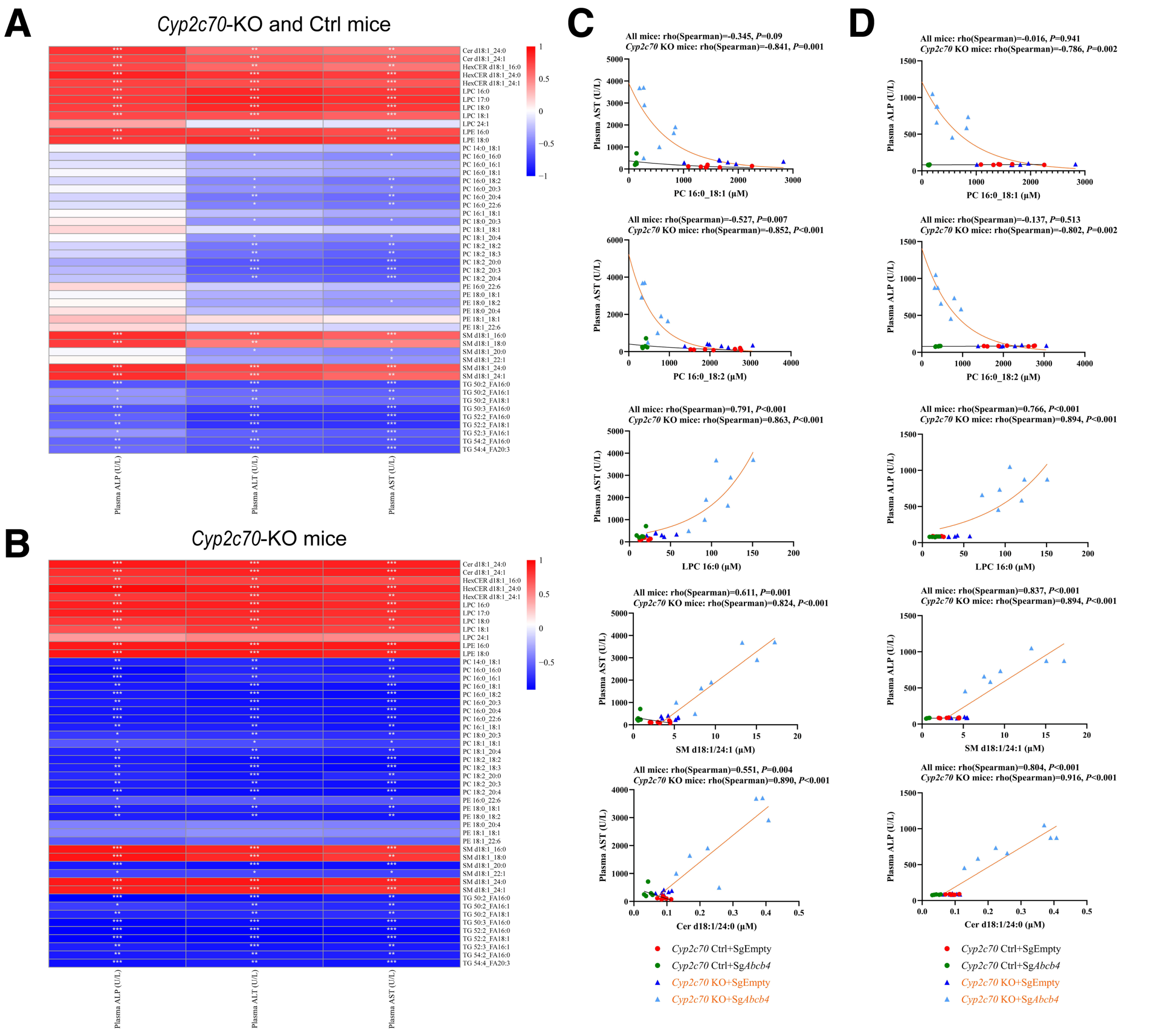

Figure 4. Associations between differentially changed biliary lipid species and liver damage markers were analyzed at 10 days after virus injection. The correlation heatmap between top 50 most significant differentially biliary lipids and liver damage markers in all mice (*A*) and *Cyp2c70* KO mice (*B*). Individual lipid species are indicated on the right. *Red*: positive correlation; *Blue*: negative correlation. Associations between biliary PC, LPC, SM, ceramide, and plasma AST (*C*), ALP (*D*) levels. **P* < .05, ***P* < .01, ****P* < .001, according to Spearman's rank correlation test. Cer, ceramide.

plasma ALT and AST, as well as ALP levels, that were mainly driven by the *Cyp2c70-KO* groups, particularly by the *Cyp2c70-KO/Abcb4-KD* mice (Figures 3*K* and 4). Indeed, biliary PC in the control mice did not show a clear association with liver enzymes in the plasma, whereas a negative relationship was clearly present in the *Cyp2c70-KO/Abcb4*-KD mice. Intriguingly, opposite relationships were found for lysoPC and "aspecific" biliary phospholipids such as ceramide and SM. Again, these relationships were mainly driven by *Cyp2c70-KO/Abcb4-KD* mice (Figures 3*L* and 4). These data show that the relative contributions of "bile-specific" (ie, PC) and "bile-aspecific" PL in bile reflect the severity of liver disease in PFIC3 mice with a human-like BA composition. These observations are consistent with the concept that the nonselective extrusion of PL from membranes by unsaturated hydrophobic BA micelles contributes to disease development.

Prolonged Abcb4 Inactivation in Cyp2c70-KO Mice Is Associated With Progression of Liver Pathology

Although marked liver pathology was already apparent in *Cyp2c70*-KO mice at 10 days after injection with the AAV-sg*Abcb4* virus, histological alterations were mainly confined to the portal areas at this relatively early time point. To explore the longer-term effects of *Abcb4* inactivation on liver pathology in *Cyp2c70*-KO mice, a second cohort of animals was sacrificed 6 weeks after injection of AAV-

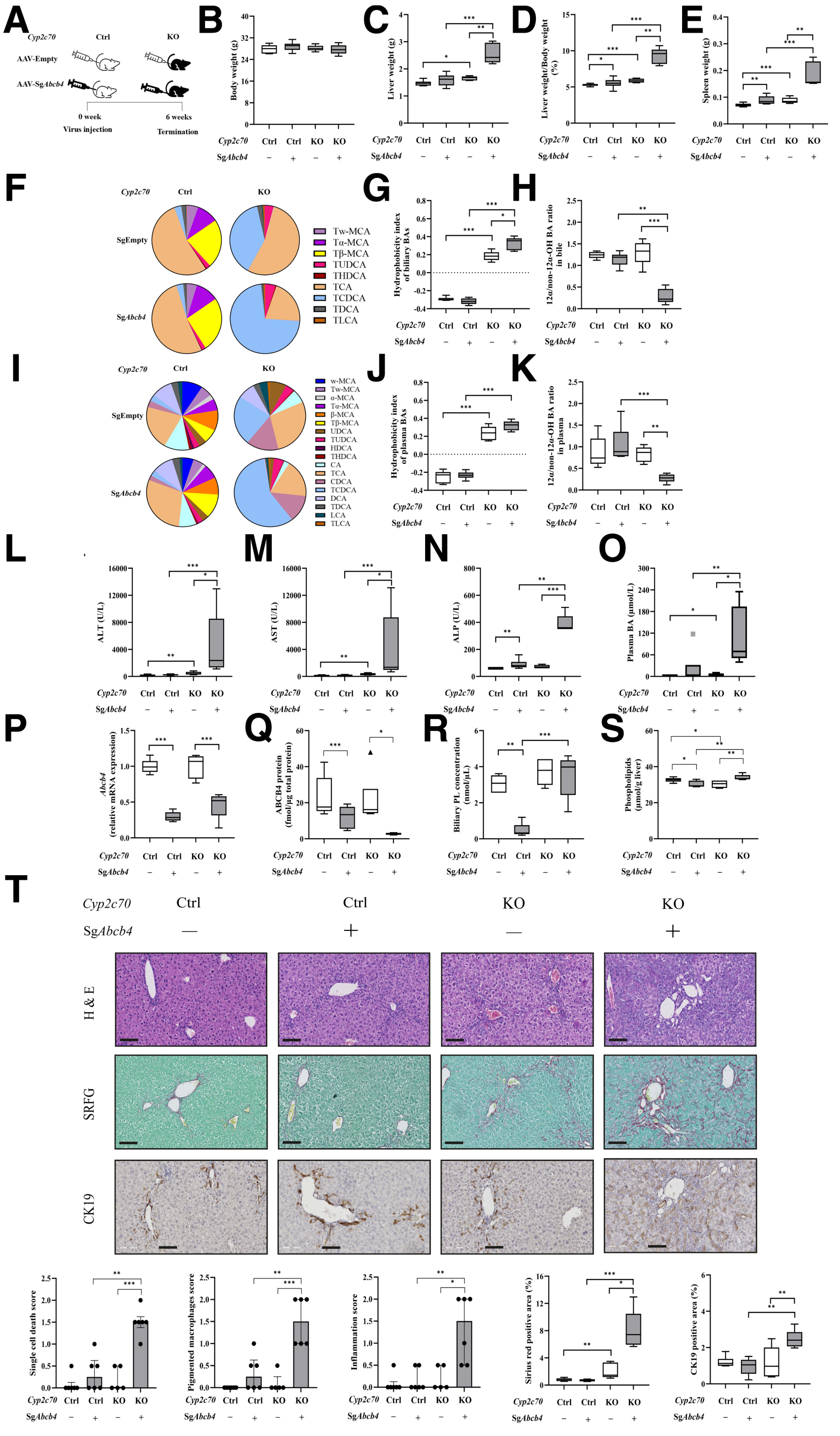

Figure 5. Advanced liver pathology in long-term *Cyp2c70*-KO/*Abcb4*-KD mice. (*A*) Study design: male control and *Cyp2c70*-KO mice (n = 5–6 per group) were injected with AAV-Empty or AAV-sg*Abcb4* and sacrificed after 6 weeks. (*B*) Body weight. (*C*) Liver weight. (*D*) The ratio of liver weight to body weight. (*E*) Spleen weight. (*F*) Biliary BA profile. (*G*) Hydrophobicity index of biliary BAs. (*H*) Biliary 12α-/non12α-OH BA ratio. (*I*) Plasma BA profile. (*J*) Hydrophobicity index of plasma BAs. (*K*) Plasma 12α-/non12α-OH BA ratio. (*L–O*) plasma transaminases (ALT, AST), ALP, and total plasma BA levels. (*P–S*) Hepatic *Abcb4* mRNA and protein expression, biliary, and hepatic PL contents. (*T*) Representative images and quantification of liver sections stained with H&E, SRFG, and anti-CK19 antibody (bars represent 100 μm). *$P < .05$, **$P < .01$, ***$P < .001$, according to Kruskal-Wallis H test followed by Conover post-hoc comparisons.

sg*Abcb4* or AAV-Empty (Figure 5*A*). After this prolonged period of *Abcb4*-KD, marked increases in liver and spleen weights were observed in *Cyp2c70*-KO/*Abcb4*-KD mice, whereas body weight was similar in all groups (Figure 5*B–E*). The ratio of 12α/non-12α-hydroxylated BAs was markedly decreased in bile and plasma (Figure 5*F–K*), and the hydrophobicity index of biliary BAs tended to be even higher when *Abcb4* was inactivated in *Cyp2c70*-KO mice for 6 weeks compared with 10 days (ie, 0.3 vs 0.2; $P = .073$, respectively) (Figures 1*H* and 5*G*). Accordingly, plasma

transaminases, ALP, and total plasma BA levels were strongly elevated in *Cyp2c70*-KO/*Abcb4*-KD mice compared with those in control/*Abcb4*-KD mice (Figure 5*L*–*O*), delineating the importance of hydrophobic, human-like BA composition in aggravating liver injury upon *Abcb4* inactivation. Gene expression of *Abcb4*, ABCB4 protein content and the biliary PL concentrations were still reduced in control mice at 6 weeks after *Abcb4*-KD, whereas biliary PL concentrations were similar in *Cyp2c70*-KO mice injected with AAV-sg*Abcb4* or AAV-Empty despite reduced *Abcb4* gene and ABCB4 protein expression (Figure 5*P*–*R*). Hepatic PL content was slightly higher in Cyp2c70-KO mice injected with AAV-sgAbcb4 than in those injected with AAV-Empty (Figure 5*S*). Histological evaluation revealed of the livers that *Cyp2c70*-KO/*Abcb4*-KD mice had higher incidence of single-cell death, more pigmented macrophages, and increased inflammation score compared with control/*Abcb4*-KD mice (Figure 5*T*). In addition, fibrosis had become bridging at this time point, and ductular reactions were prominently present in *Cyp2c70*-KO/*Abcb4*-KD mice (Figure 5*T*), demonstrating the progression of liver pathology between 10 days and 6 weeks post-inactivation of the *Abcb4* gene.

Hepatic Gene Expression Patterns Indicate Induction of Epithelial Mesenchymal Transition in Cyp2c70-KO/Abcb4-KD Mice

To gain insight into the cellular processes that might contribute to the development and progression of liver pathology in *Cyp2c70*-KO/*Abcb4*-KD mice, transcriptome analysis was performed. Principal component analysis (PCA) fully separated the *Abcb4*-KD mice from the mice injected with AAV-Empty virus. Importantly, *Cyp2c70*-KO/*Abcb4*-KD mice showed clear separation from control/*Abcb4*-KD mice, indicating that gene expression patterns differ between the ‘humanized’ and the ‘conventional’ PFIC3 mouse model (Figure 6*A*). *Cyp2c70*-KO/*Abcb4*-KD mice were separated from all other groups mainly on PC1. The most upregulated genes impacting this PC were *Ly6d*, *Sprr1a*, *Tspan8*, *Mmp7*, and *Lcn2*, while the most downregulated genes consisted of members of the major urinary protein (MUP) family, but also of genes involved in BA metabolism (ie, *Hsd3b5* and *Cyp8b1*) (Figure 6*B*). MUP family proteins are involved in pheromone-based communication in mice,[34] and their reduced expression likely reflects an adaptive response to hepatic stress upon hepatic *Abcb4* inactivation.[35] Gene set enrichment analysis (GSEA) using mouse-ortholog hallmark gene sets revealed that epithelial mesenchymal transition (EMT) was most upregulated (NES = 3.126; $P_{adj} = .004$) in *Cyp2c70*-KO/*Abcb4*-KD mice compared with control/*Abcb4*-KD mice (Figure 6*C* and *D*). In line, the transcriptomics data also indicated that transforming growth factor-β (TGF-β) signaling, a major promoter of EMT,[36] was induced in *Cyp2c70*-KO/*Abcb4*-KD mice as indicated by pathway analysis, with upregulation of genes encoding key ligands (*Tgfb*, *Bmps*), receptors (*Tgfbr1/2*, *Bmpr1*), and downstream effectors such as *Smad2/3*, *Myc* and *p15* (Figure 6*E*). In addition, upregulation of the transcriptional regulators *Snai1* and *Zeb2* support ongoing EMT (Figure 6*F*).[36] The presence of cells that stained positive for the EMT marker *S100a4*[37–39] using fluorescent in situ hybridization (FISH) of RNA only in *Cyp2c70*-KO/*Abcb4*-KD mice provides further support for the activation of this process upon knockdown of *Abcb4* in the context of a human-like BA composition (Figure 6*G*). In addition to EMT, gene sets encompassing angiogenesis, cell cycle regulation, and inflammatory pathways were highly upregulated upon *Abcb4*-KD in *Cyp2c70*-KO mice compared with its inactivation in control mice. Metabolic pathways (oxidative phosphorylation, fatty acid metabolism, and BA metabolism) were among the most downregulated processes in *Abcb4*-KD mice with human-like BA composition, indicating pronounced effects on liver function.

Colesevelam Mitigates Liver Pathology in Male Cyp2c70-KO/Abcb4-KD Mice

Modulating the enterohepatic circulation of BAs has been reported to ameliorate cholestasis and bile duct injury in *Abcb4*-KO mice[29,40] as well as in *Cyp2c70*-KO mice.[30,41] In this study, we investigated whether BA sequestration can improve the severe liver pathology in *Cyp2c70*-KO/*Abcb4*-KD mice. Male *Cyp2c70*-KO mice, injected with AAV-Empty or AAV-sg*Abcb4*, were fed a regular chow diet or a chow diet supplemented with 2% (w/w) colesevelam from the moment of injection onwards for 6 weeks (Figure 7*A*). Body weight was similar in all groups, while addition of colesevelam to the diet did not negatively affect food intake (Figure 7*B* and *C*). The increase of liver and spleen weights induced by *Abcb4*-KD in *Cyp2c70*-KO mice were mitigated by colesevelam treatment (Figure 7*D*–*F*). In line with its function as a BA sequestrant, colesevelam treatment markedly increased fecal BA excretion in *Cyp2c70*-KO mice with and without *Abcb4* deficiency (Figure 7*G*), indicating that colesevelam effectively reduced intestinal BA absorption efficiency in these mice. Accordingly, bile flow (Figure 7*H*) and, in particular, biliary BA secretion (Figure 7*I*) were substantially decreased upon colesevelam treatment. Biliary PL secretion was reduced in colesevelam-fed mice without *Abcb4* inactivation due to impaired BA excretion, as evidenced by unaltered PL to BA ratios (Figure 7*J* and *K*), whereas decreased ratios of PL to BA were observed in *Cyp2c70*-KO/*Abcb4*-KD mice fed control chow as well as in those treated with colesevelam (Figure 7*K*). Increased ratios of 12α-/non-12α-hydroxylated BAs (Figure 7*L* and *M*) in the bile of mice treated with colesevelam translated into a tendency towards a reduction in the hydrophobicity index of biliary BAs (Figure 7*N*). A similar trend was observed in plasma BA profiles (ie, higher ratio of 12α-/non-12α-hydroxylated BAs and less hydrophobic BAs) (Figure 7O–*Q*), indicating that the circulating BA pool became somewhat less hydrophobic after colesevelam treatment.

Liver damage markers ALT, AST, and ALP that were strongly elevated in *Cyp2c70*-KO/*Abcb4*-KD mice compared with AAV-Empty injected *Cyp2c70*-KO mice were robustly reduced upon colesevelam treatment (Figure 8*A*–*C*). Likewise, the elevated plasma BA concentrations observed in

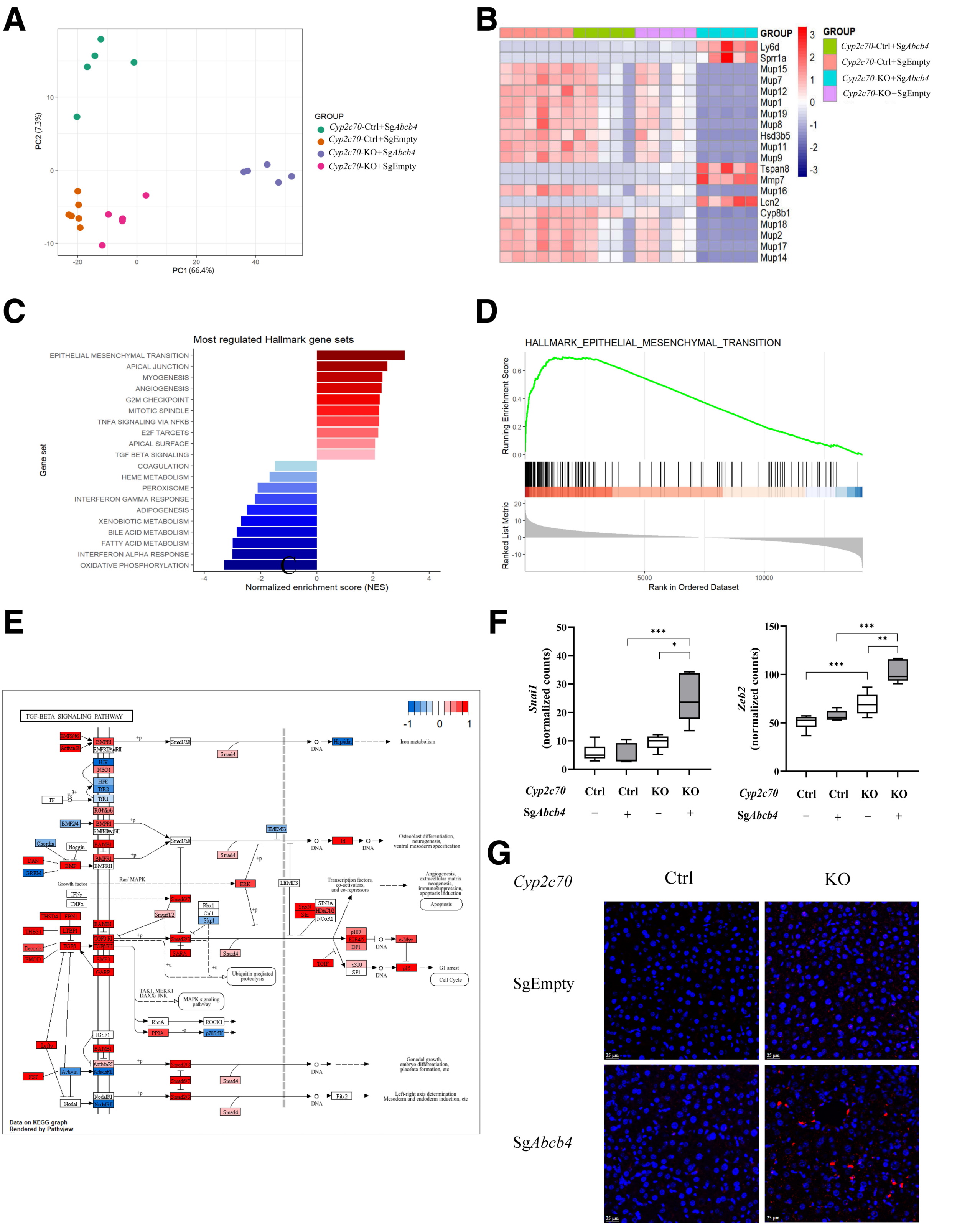

Figure 6. Genes related to EMT are upregulated in liver of *Cyp2c70*-KO mice upon *Abcb4* ablation. (*A*) PCA of gene expression profiles. (*B*) Top 20 genes impacting PC1. (*C*) GSEA analysis of hallmark gene sets between livers of *Cyp2c70*-KO/*Abcb4*-KD and Ctrl/*Abcb4*-KD mice (gene sets with a Benjamini-Hochberg adjusted *P* value $<$ .05 were considered significantly enriched). (*D*) GSEA enrichment score plot of EMT. (*E*) Overview of gene expression changes in the KEGG TGF-β signaling pathway, generated using the Pathview package for R. *Red*: upregulated genes; *Blue*: downregulated genes. (*F*) RNA expression of *Snai1* and *Zeb2*. (*G*) Representative SABER TSA FISH staining of *S100a4* mRNA in liver sections. *Red*: *S100a4* mRNA; *Blue*: DAPI staining for nuclei (bars represent 25 μm). *$P <$ .05, **$P <$ .01, ***$P <$ .001, according to Kruskal-Wallis H test followed by Conover post-hoc comparisons.

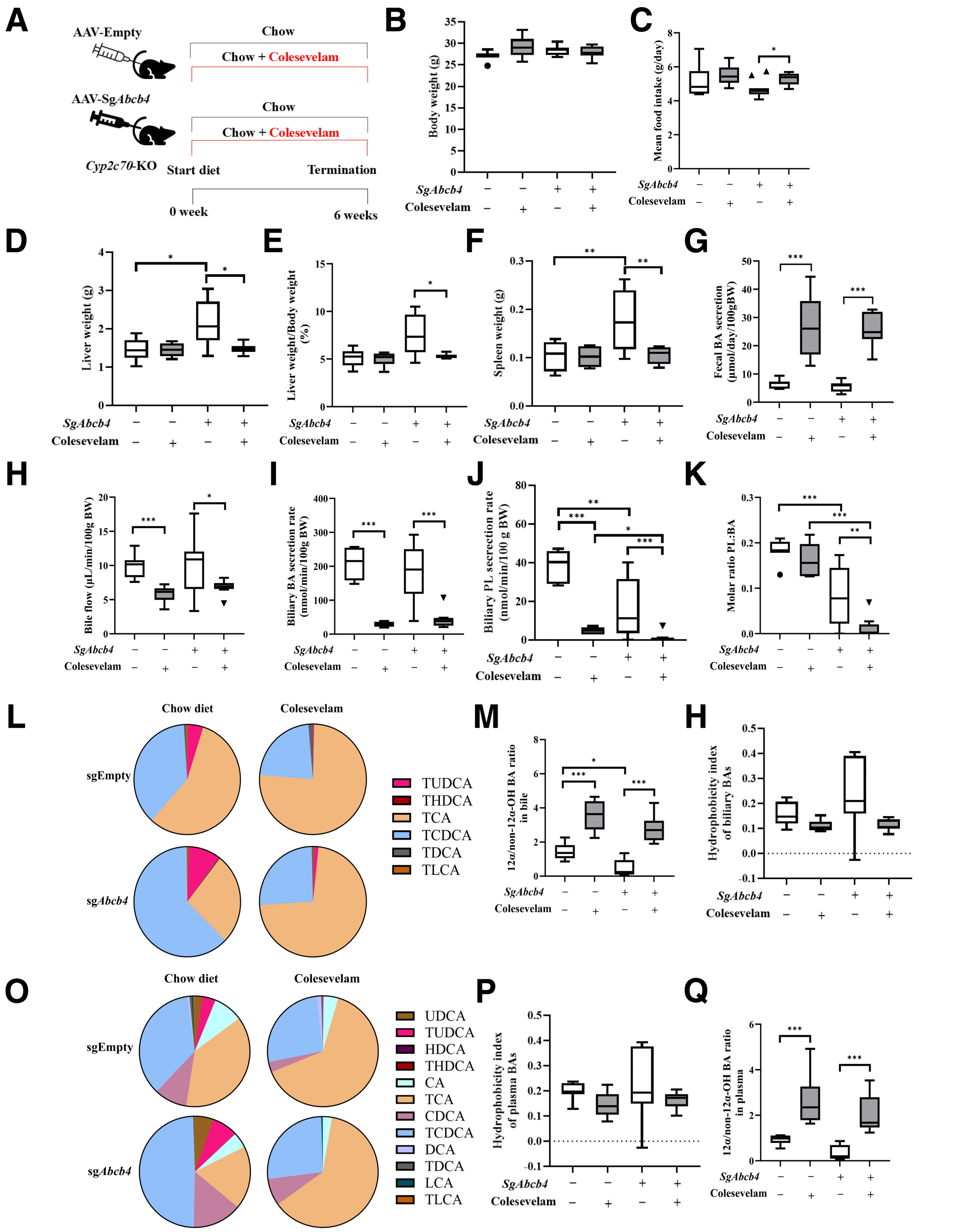

Figure 7. Colesevelam reduces biliary BA secretion and alters BA composition in *Cyp2c70*-KO/*Abcb4*-KD mice. (*A*) Study design: male *Cyp2c70*-KO/L-cas9tg mice were injected with AAV-Empty or AAV-sg*Abcb4* and maintained on a regular chow diet with or without colesevelam for 6 weeks (n = 6–9 per group). (*B*) Body weight. (*C*) Mean food intake. (*D*) Liver weight. (*E*) The ratio of liver weight to body weight. (*F*) Spleen weight. (*G*) Fecal BA excretion. (*H*) Bile flow. (*I*) Biliary BA secretion. (*J*) Biliary PL secretion. (*K*) PL:BA molar ratios. (*L*) Biliary BA profile. (*M*) Biliary 12α-/non-12α-hydroxylated BA ratio. (*N*) Hydrophobicity index of biliary BAs. (*O*) Plasma BA profile. (*P*) Hydrophobicity index of plasma BAs. (*Q*) Plasma 12α-/non12α-OH BA ratio. *$P < .05$, **$P < .01$, ***$P < .001$, according to Kruskal-Wallis H test followed by Conover post-hoc comparisons.

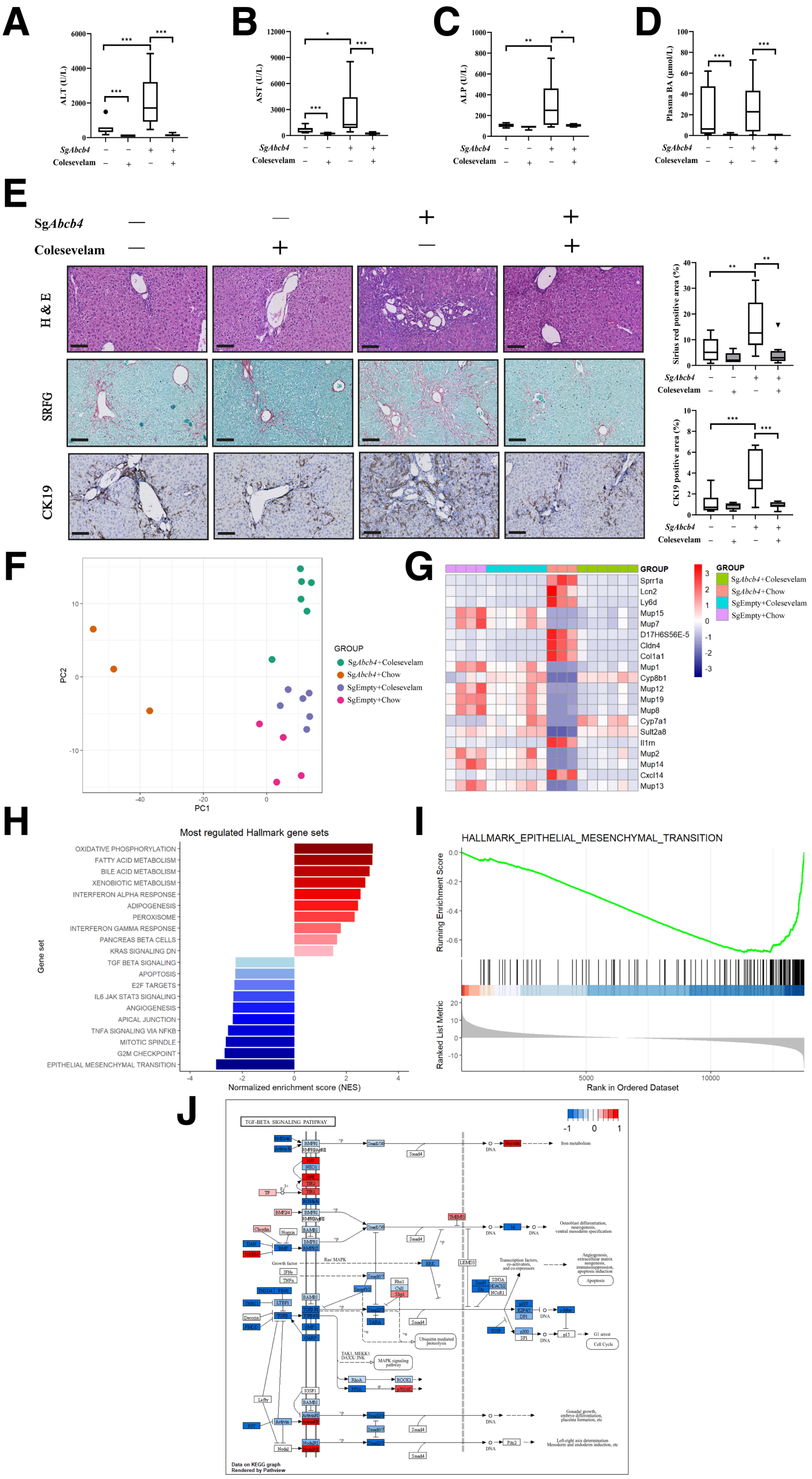

Figure 8. Colesevelam ameliorates liver pathology in *Cyp2c70*-KO/*Abcb4*-KD mice. (*A–D*) Plasma transaminases (ALT, AST), ALP, and total BA levels. (*E*) Representative images and quantification of liver sections stained with H&E, Sirius Red, and anti-CK19 antibody (bars represent 100 μm). (*F*) PCA of gene expression profiles. (*G*) Top 20 genes impacting PC1. (*H*) GSEA analysis of Hallmark gene sets between livers of colesevelam-treated and untreated *Cyp2c70*-KO/*Abcb4*-KD mice (gene sets with a Benjamini-Hochberg adjusted *P* value < .05 were considered significantly enriched). (*I*) GSEA enrichment score plot of EMT. (*J*) Overview of gene expression changes in the KEGG TGF-β signaling pathway, generated using the Pathview package for R. *Red*: upregulated genes; *Blue*: downregulated genes. *$P < .05$, **$P < .01$, ***$P < .001$, according to Kruskal-Wallis H test followed by Conover post-hoc comparisons.

both *Cyp2c70*-KO and *Cyp2c70*-KO/*Abcb4*-KD mice strongly decreased upon colesevelam treatment (Figure 8*D*), whereas augmented collagen deposition and ductular reactions induced by *Abcb4* inactivation in the livers of *Cyp2c70*-KO mice were fully reversed by colesevelam treatment (Figure 8*E*). To gain insight into the hepatocellular processes

affected by the interruption of the enterohepatic circulation of BAs by colesevelam, we performed bulk RNA sequencing (RNA-seq) on the livers of the respective groups. PCA analysis revealed a clear separation between colesevelam-treated and untreated *Cyp2c70*-KO/*Abcb4*-KD mice, mainly on PC1 (Figure 8*F*). Liver gene expression patterns impacting this PC indicated that colesevelam treatment normalized the aberrant gene expression profile observed upon *Abcb4*-KD in *Cyp2c70*-KO mice (eg, *Sprr1a*, *Ly6d* and *Lcn2* were downregulated, while *Cyp8b1* and MUP family genes were upregulated) (Figure 8*G*). Interestingly, GSEA suggested reduced EMT and TGF-β signaling in colesevelam-treated vs untreated *Cyp2c70*-KO/*Abcb4*-KD mice (Figure 8*H–J*). The Kyoto Encyclopedia of Genes and Genomes (KEGG) TGF-β pathway map shows that colesevelam treatment generally reduced the expression of genes encoding key ligands (*Tgfb*, *Bmps*), receptors (*Tgfbr1/2*, *Bmpr1/2*), and downstream effectors such as *Smad2/3* and *Myc* (Figure 8*J*). Together, these data demonstrate that interruption of enterohepatic circulation by colesevelam ameliorates the severe liver pathology induced by *Abcb4* inactivation in *Cyp2c70*-KO mice with a human-like BA composition.

Discussion

This study reveals that suppression of hepatic *Abcb4* gene expression in the context of a human-like BA composition (ie, in *Cyp2c70*-KO mice) induces much more pronounced liver pathology as compared with *Abcb4* inactivation in the context of a normal murine BA composition rich in hydrophilic MCAs. Pathological features that were more prominent in *Cyp2c70*-KO mice than in control mice upon *Abcb4*-KD include hepatocyte damage, inflammation, bile duct proliferation, and severe bridging fibrosis, whereas biliary BA secretion rates and bile production per se remained unaffected. The biliary lipidome analysis confirmed a decrease in PC upon *Abcb4* suppression in both *Cyp2c70*-KO and control mice. Of particular note, the abundances of several "aspecific" lipid species in bile, such as lysoPC 16:0 and SM d18:1/24:1, were strongly increased in *Cyp2c70*-KO mice only upon *Abcb4*-KD and strongly associated with liver damage. These findings indicate that hydrophobic BAs in undersaturated mixed micelles induce lipid extrusion from the canalicular membranes of hepatocytes and/or the apical membranes of cholangiocytes, thereby causing severe liver pathology in *Cyp2c70*-KO/*Abcb4*-KD mice. This mode of action is supported by the observation that the development of liver pathology upon suppression of *Abcb4* in *Cyp2c70*-KO mice could be effectively counteracted by BA sequestration with colesevelam, which reduced biliary BA concentrations and decreased the contribution of hydrophobic CDCA in the circulating BA pools of the treated mice.

The generation of *Abcb4* KO mice on a FVB background in 1993[5] led to the identification of ABCB4, at the time referred to as MDR2 in mice and MDR3 in humans, as an ATP-dependent transporter localized at the hepatocyte canalicular membrane responsible for PC translocation from the inner to the outer leaflet and, ultimately, into bile. Subsequent studies revealed that the rate of biliary PC secretion is directly related to the expression of the *Abcb4* gene in mice (ie, reduced by ~50% in *Abcb4*$^{+/-}$ mice[5,42] and restored to WT rates upon AAV-mediated gene transduction into *Abcb4*-KO mice).[14] It was also noted that *Abcb4*-KO mice have an increased bile flow, due to a higher BA-dependent as well as a higher BA-independent bile flow,[43] show bile duct proliferation,[5] and relatively slowly develop biliary fibrosis during adulthood.[20,44] These features differ from those observed in human PFIC3, in which cholestatic liver disease progresses rapidly and severely in early life, and liver transplantation is often needed for survival already during childhood.[2] In fact, *Abcb4*-KO mice have more recently been advocated as a model for PSC[19] rather than for PFIC3. Our data indicate that this is at least partly attributable to the lower "cytotoxicity" of murine bile, rich in hydrophilic MCAs, compared with human bile. In this study, we aimed to develop a more representative PFIC3 model by inactivating *Abcb4* in *Cyp2c70*-KO mice with a human-like BA composition. Our model reflects a state of partial *Abcb4* deficiency rather than a complete *Abcb4*-null state. Importantly, the minor residual ABCB4 function in combination with a human-like BA profile replicates key pathogenic features of human PFIC3. Several disease-causing ABCB4 missense variants identified in PFIC3 patients (eg, S242R, S346I, T437I, T1077M) have been shown to traffic correctly to the canalicular membrane and to display residual PC transport activity. Importantly, subjects expressing these variants show sensitivity to CFTR potentiators.[45] Therefore, this humanized PFIC3 mouse model allows us to evaluate potential tailored treatment strategies for patients with PFIC3 and some residual ABCB4 activity, such as interruption of the enterohepatic circulation of BAs and potentiation of (residual) ABCB4 function,[46] whereas *Cyp2c70*/*Abcb4*-DKO mice would reflect the situation in patients with PFIC3 without residual ABCB4 activity and may represent the preferred model to evaluate the efficacy of gene or mRNA therapy approaches.

It is important to note that, despite the evident signs of liver damage, *Cyp2c70*-KO/*Abcb4*-KD mice do not fulfill the classical definition of cholestasis, which typically involves impaired bile flow. This is a feature shared by all PFIC models published so far (eg, PFIC1 [ATP8B1-deficiency],[47] PFIC2 [BSEP-deficiency],[48] and PFIC5 [FXR-deficiency][49]) and indicates the presence of intrinsic differences in the bile formation process between mice and humans. Nevertheless, plasma transaminases were somewhat elevated in control/*Abcb4*-KD but much more markedly and progressively in *Cyp2c70*-KO/*Abcb4*-KD mice, indicative for more pronounced hepatic injury in the latter. In addition, *Cyp2c70*-KO/*Abcb4*-KD mice developed severe cholangiopathy with bridging fibrosis within a 6 weeks' time frame, with histological features similar to those seen in the livers of patients with PFIC3,[1,2] which did not occur in control/*Abcb4*-KD mice. This difference is conceivably attributable to the hydrophobic, human-like BA pool in *Cyp2c70*-KO mice, which is rich in CDCA and lacks MCAs. The altered BA profile in *Cyp2c70*-KO mice is associated with an age-dependent development of cholangiopathy and liver fibrosis in

females, whose BA pool is enriched in CDCA compared with those of males due to low expression of hepatic *Cyp8b1*.[23,25] This conclusion is supported by the observation that colesevelam treatment, leading to significantly lower biliary BA concentrations with smaller contributions of CDCA, prevents hepatocellular damage as well as fibrosis development in *Cyp2c70*-KO/*Abcb4*-KD mice, even though the total biliary PL content was strongly reduced under these conditions.

Assessment of the biliary lipidome confirmed that *Abcb4*-KD decreased the concentrations of bile-specific PC species, such as PC 16:0_18:1, PC 16:0_18:,2 and PC 16:0_20:4, in the bile of both *Cyp2c70*-KO and control mice. The specificity of biliary PC composition is governed by the actions of ABCB4, whereas the rate of biliary PC secretion is determined by a combination of ABCB4 activity and biliary BA hydrophobicity and secretion rate.[50] Indeed, increasing biliary BA secretion in conditions with a fixed *Abcb4* expression level will promote biliary PC secretion up to a certain plateau level[50]; more hydrophobic BA species display a higher capacity to stimulate biliary PC secretion than more hydrophilic species.[51] Consequently, the biliary PL/BA ratios in *Cyp2c70*-KO mice are higher than those in the respective WT controls under basal conditions.[25] Once inside the bile canalicular space, BAs, PC and cholesterol form mixed micelles. When sufficiently saturated and stabilized, these micelles likely prevent the detergent actions of local millimolar BA concentrations on the canalicular membranes of hepatocytes as well as the apical membranes of cholangiocytes and ductular cells. Thereby, micelle formation provides a form of protection that prevents injury of epithelial liver cells. This study and an earlier study in *Abcb4*-KO mice on the *Bsep*-KO background[52] indicate that the presence of relatively high concentrations of hydrophilic BAs, such as MCAs and tetrahydroxylated bile acids (THBAs), in murine bile reduces its toxicity towards its membranous environment and, consequently, blunts the development of liver pathology, even in the absence of adequate biliary PC concentrations.

Remarkably, the bile of *Cyp2c70*-KO/*Abcb4*-KD mice was enriched in lysoPC compared to all other groups. Biliary lysoPC is considered to be mainly derived from hydrolysis of PC by cytosolic phospholipase A_2 (cPLA$_2$), which is encoded by the *Pla2g4a* gene.[53] It has been reported that particularly CDCA is able to induce *Pla2g4a* expression in human alveolar epithelial cells by inducing phosphorylation of mitogen-activated protein kinases (MAPKs).[54] Indeed, gene expression analyses revealed that the expression of *Pla2g4a* was upregulated in the livers of *Cyp2c70*-KO/*Abcb4*-KD mice. A similar induction of hepatic *Pla2g4a* expression was, however, observed in control/*Abcb4*-KD mice that did not have high amounts of CDCA in their circulating BA pools, nor did these mice have high abundances of lysoPC in their bile. Thus, the exact origin of the lysoPC present in the bile of *Cyp2c70*-KO/*Abcb4*-KD mice remains to be established. Irrespective of the way it is generated, lysoPC is known to drive lymphocyte and macrophage migration, enhance proinflammatory cytokine production, induce oxidative stress, and trigger apoptosis,[55–57] and thereby may exacerbate pathology in the PFIC3 model described in the current study. From a mechanistic perspective, the increased proportions of ceramide, SM, and lysoPC and their strong relationships with plasma markers of liver damage are of particular interest. ABCB4 primarily transports PC but has been reported to mediate the efflux of SM and phosphatidylethanolamines (PE).[58,59] This may explain the relatively low SM and PE levels in the bile of control/*Abcb4*-KD mice compared with control mice that received AAV-Empty. However, the markedly increased presence of these “bile-aspecific” PL in the bile of *Cyp2c70*-KO/*Abcb4*-KD mice is likely a consequence of improperly ‘shielded’ hydrophobic BAs within the canaliculi and bile ductules that extract these PL from the surrounding cell membranes.[60] The appearance of bile-aspecific PL has also been described during the infusion of BAs at supraphysiological rates and, in these experiments, to precede the induction of cholestasis,[61] supporting the notion that the presence of these PL in bile is indicative of hepatocellular and/or cholangiocyte damage. It is likely that the comparable PL/BA ratios between *Cyp2c70*-KO/*Abcb4*-KD mice and *Cyp2c70*-KO mice at 6 weeks after virus injection reflects PL solubilization from membranes by hydrophobic BAs since *Abcb4* expression was still strongly reduced at this time point. This concept is further supported by the observation that treatment of *Cyp2c70*-KO/*Abcb4*-KD mice with the BA sequestrant colesevelam led to a normalization of plasma ALT, AST, ALP, and BA levels, whereas biliary PL was fairly low. We propose that this is related to the low biliary BA concentrations of 5 to 6 mM with a concomitant shift in BA synthesis towards increased production of more hydrophilic CA relative to CDCA driven by enhanced sterol 12α-hydroxylase activity due to *Cyp8b1* upregulation.

The current study provides the first clue regarding the mechanism(s) underlying the unprecedentedly rapid development of fibrosis in male *Cyp2c70*-KO/*Abcb4*-KD mice: distinct fibrosis was observed at 10 days after *Abcb4*-KD, whereas massive bridging fibrosis developed within a 6 weeks’ time frame. Interestingly, GSEA on RNA-seq data revealed that EMT was the most upregulated Hallmark gene set in the fibrotic livers of *Cyp2c70*-KO/*Abcb4*-KD mice compared with the non-fibrotic livers of control/*Abcb4*-KD mice, indicating the relevance of the presence of a human-like BA composition herein. EMT has been proposed as a mechanistic player in the development of liver fibrosis.[36] This process allows hepatocytes and cholangiocytes to acquire specific mesenchymal features, including the ability to produce extracellular matrix components, such as collagens. EMT is considered to be driven by profibrotic cytokines such as TGF-β.[36,62,63] Indeed, the expression of many components of the TGF-β signaling pathway was strongly upregulated in the livers of *Cyp2c70*-KO/*Abcb4*-KD mice, suggesting increased activity of this pathway. Moreover, the expression levels of *Snai1* and *Zeb2*, which encode major transcription factors involved in EMT,[36] were increased mainly in *Cyp2c70*-KO/*Abcb4*-KD mice. With FISH, we were able to demonstrate a clearly higher abundance of cells expressing *S100a4* mRNA, a universally used marker of EMT-derived cells,[37–39] in livers of *Cyp2c70*-KO/

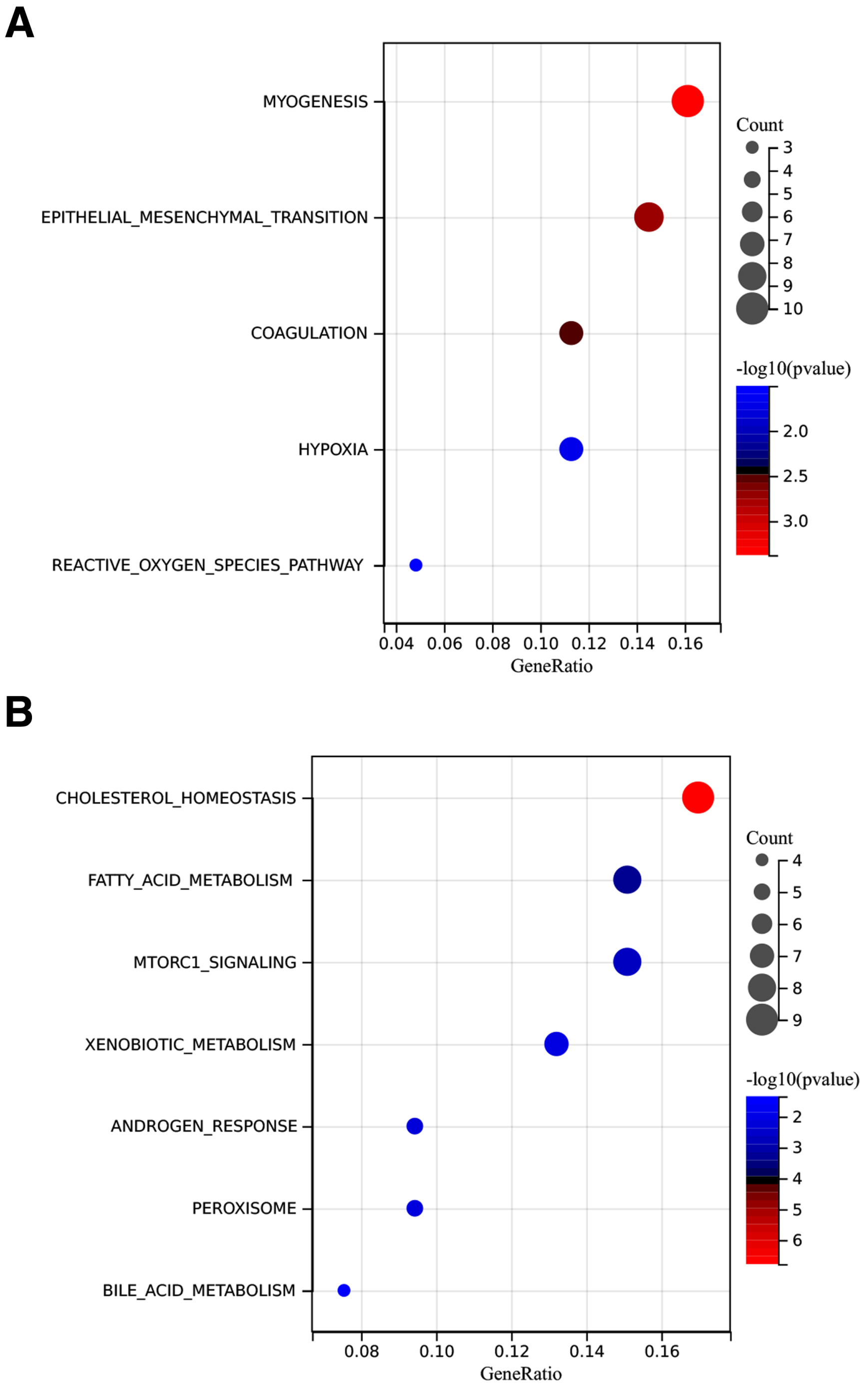

Figure 9. Proteins involved in EMT are upregulated (Benjamini-Hochberg adjusted *P* value = .03) in patients with PFIC3 compared with healthy controls. Hallmark gene sets enrichment analysis of significant upregulated (*A*) and downregulated (*B*) proteins between PFIC3 livers and control livers.

Abcb4-KD mice than in any of the other groups. Of note, reduction of fibrosis of *Cyp2c70*-KO/*Abcb4*-KD mice by colesevelam treatment was associated with strong downregulation of EMT-related genes as well as genes involved in the TGF-β signaling pathway, supporting the existence of an association between fibrogenesis and EMT in our PFIC3 model. Intriguingly, examination of a publicly available proteomics dataset of livers from human patients with PFIC3[64] indicated that proteins involved in EMT were highly upregulated in these individuals compared with healthy controls (Figure 9), delineating the potential translational relevance of these findings. The quantitative relevance of EMT to the myofibroblast population contributing to fibrosis development in human liver diseases, however, has been heavily debated,[37] and it has been postulated that EMT may reflect a response to the fibrotic and inflammatory environment rather than a causal event.[65] Such an environment is obviously present in the livers of *Cyp2c70*-KO/*Abcb4*-KD mice, as well as in those of patients with PFIC3.

While this manuscript was in preparation, Tsuruya et al[66] reported that CYPDKO (*Cyp2c70/Cyp2a12* double

knockout)/*Abcb4*-KD mice also show ductular reactions, liver fibrosis, and increased plasma transaminases, but not increased plasma BAs, compared with WT/*Abcb4*-KD mice, which basically aligns with our findings. However, there are some fundamental differences between their study and the present work, apart from the fact that CYPDKO has a BA pool enriched in secondary BAs. First, our PFIC3 mouse model appears to display more severe liver damage than CYPDKO/*Abcb4*-deficient mice, according to the provided values of plasma transaminases and BA concentrations. Interestingly, Tsuruya et al did not quantify ABCB4 protein levels and showed only ∼50% KD efficiency on *Abcb4* mRNA levels in their CYPDKO-mice, whereas *Abcb4* mRNA level was not affected in WT mice upon *Abcb4*-KD. Differences in gene ablation procedures (ie, injection of a single sgRNA-containing virus into *Cyp2c70*-KO/L-Cas9tg mice vs injection with 2 separate viruses, one containing a SaCas9-encoding DNA sequence and the other one encoding 3 different *Abcb4*-targeting gRNAs)[66] may have impacted the development of liver pathology. In addition, early and longer-term time points were included in our current study. This longitudinal characterization allows us to capture disease progression over time. Furthermore, our study provides important mechanistic insights based on lipidomics, transcriptomics, and SABER procedures. This multi-layered approach mechanistically links specific changes in biliary lipid composition and key biological processes to hepatobiliary injury. Importantly, we demonstrate for the first time that BA sequestration with colesevelam effectively ameliorates liver pathology in this PFIC3 model by lowering biliary BA concentrations, thereby establishing its value as a preclinical model for therapeutic testing. Our data thus add substantially to the understanding of the modes of action involved in PFIC3-associated liver disease. Recent studies[41,67] have highlighted the importance hepatic BA accretion in development of the cholestatic phenotypes in Cyp2c70-KO mice. Therefore, assessment of intrahepatic BA concentrations and fluxes using newly developed techniques such as intravital microscopy[68] could further increase insight in disease development.

In conclusion, this study demonstrates that suppression of *Abcb4* gene expression in the livers of *Cyp2c70*-KO mice with a hydrophobic, human-like BA pool composition profoundly exacerbates liver pathology compared with hepatic inactivation in control mice, as evidenced by time-dependent increases in plasma liver damage markers, enhanced ductular reactions, and severe bridging fibrosis. We propose that this phenotype is driven by damage to epithelial cell membranes in the biliary tree due to the high detergency of improperly 'shielded' hydrophobic BAs. This concept is supported by the reversal of pathology upon colesevelam treatment, which is associated with reduced biliary BA concentration. We hypothesize that epithelial cell damage drives inflammation-associated EMT, which can be associated with rapid fibrogenesis. Our study indicates that modulating the concentration and composition of biliary BAs, particularly in the early phases of the disease, may slow down the progression of liver pathology in patients with PFIC3.

Materials and Methods

Animals

Cyp2c70-KO (C57BL/6J-*Cyp2c70*^{em3Umcg})[25] mice were crossbred with liver-specific Cas9-transgenic (L-Cas9tg) mice, that had been generated by crossbreeding B6.129-Gt(ROSA)26Sor$^{tm1(CAG-xstpx-cas9,-EGFP)Fezh}$/J mice (#024857, The Jackson Laboratory), backcrossed to a C57BL6/J background, with Alb-cre mice (#003574, The Jackson Laboratory) in our animal facility,[69] to generate *Cyp2c70*-KO/L-Cas9tg mice.[70] Importantly, *Cyp2c70*-HET mice have a BA composition similar to that of WT mice and are phenotypically indistinguishable.[25] Therefore, both *Cyp2c70*-HET and WT mice were used as controls in this study. As female *Cyp2c70*-KO mice are intrinsically more prone to develop cholangiopathy than their male counterparts,[25] it was anticipated that pathophysiological consequences of *Abcb4* suppression would be masked by prevailing female-specific liver disease. Therefore, only male mice were used in the current studies. Male *Cyp2c70*-KO/L-Cas9tg mice and *Cyp2c70*-control (HET and WT)/L-Cas9tg littermates of 9 to 14 weeks old ($n = 5–7$ mice per group for the 10 days experiment and $n = 5–6$ mice per group for the 6 weeks experiment) were randomized based on body weight, age, breeding pair origin, generation, and nest number. A subset of mice received an intravenous injection with 1×10^{11} genome copies of self-complementary adeno-associated virus (scAAV)[70] containing *Abcb4* sgRNAs (detailed below) in phosphate-buffered saline (PBS) for CRISPR/Cas9-mediated inactivation in hepatocytes, whereas others were injected with a control virus (AAV-Empty) not containing sgRNA. All animals were maintained in a temperature-controlled environment (21°C) under a 12-hour light/dark cycle with free access to food and water. Additionally, a separate cohort of male *Cyp2c70*-KO/L-Cas9tg mice ($n = 6–9$ mice per group), aged 11 to 15 weeks, was treated with either AAV-Empty or AAV-sg*Abcb4* and placed on a standard chow diet (Sniff 1554 R/M-H maintenance diet) supplemented with 2% (w/w) colesevelam hydrochloride (Daiichi Sankyo Pharma Development) or received the standard chow diet for 6 weeks. Bile cannulation was performed in the morning without fasting. Hepatic bile was collected continuously for 30 minutes as described previously.[25] All mice were sacrificed by cardiac puncture under isoflurane anesthesia. Plasma was collected following centrifugation at 8000 rpm for 10 minutes at 4°C and stored at −80°C until further analyses. The organs were rapidly excised and snap-frozen in liquid nitrogen. All experiments were approved by the Dutch National Committee for Animal Experiments (AVD10500202115447), and the experimental procedures were authorized by the Animal Welfare Body of the University of Groningen.

Virus Production

For each scAAV, a DNA construct was synthesized including 3 expression cassettes encoding sgRNA sequences against *Abcb4* (5′-AAACGGAACAGCACGGCGCC-3′; 5′-CTAGTTCAAAGTCGCCGTCC-3′; 5′-CTGGACGGCGACTTT

GAACT-3′) (Thermo Fisher). These were inserted into a scAAV2 backbone vector using Kpnl and Notl restriction enzymes (New England Biolabs), and the sequences were verified. Using polyethylenimine (PEI; 23966-2, Polysciences Inc), HEK293T cells encoding the adenovirus early region 1 (E1) were transfected with 3 plasmids: the backbone vector, a helper plasmid (pAd-deltaF6, #112867, Addgene; a gift from James M. Wilson), and a Rep/Cap-encoding plasmid (pAAV2/8, Addgene; #112864; a gift from James M. Wilson). The produced virus particles were isolated using an iodixanol gradient and concentrated by spin filter centrifugation with a 100 kD cutoff (UFC910024, Merck). The number of purified AAV genome copies was quantified using quantitative polymerase chain reaction (qPCR), and virus aliquots were stored at −80°C until use.

Plasma Chemistry

The concentrations of plasma ALT, AST, and ALP were assessed using a COBAS 6000 clinical chemistry analyzer (Roche Diagnostics) with standard reagents.

BA Measurements

Fecal samples were collected over a 48-hour period, dried, and thoroughly ground. Approximately 30 mg of each sample was used for BA extraction. Samples were heated in 2 mL alkaline methanol at 80°C for 3 hours, cooled down, and ultrasonicated for 15 minutes. The mixture was then vigorously shaken for an additional 15 minutes prior to centrifugation at 3000 × g for 10 minutes. Next, 25 μL of the supernatant was used for solid-phase extraction with Oasis HLB Cartridges (Waters), prior to which deuterium-labeled internal standards were added. BAs present in plasma, bile, and feces were quantified using an ultra-high performance liquid chromatography-tandem mass spectrometry (UHPLC-MS/MS) system. The analysis was performed on an ACQUITY Premier UPLC system (Waters) coupled to a Xevo TQ-S micro triple quadrupole mass spectrometer (Waters). Stable isotope-labeled internal standards were employed for precise quantification, following previously established protocols.[25] The hydrophobicity index of biliary BAs was determined according to the method described by Heuman.[71]

Biliary Lipid Measurements

Biliary PL concentration was determined as previously described,[72] following lipid extraction according to Bligh and Dyer.[73]

Histological Examination

A section of the large liver lobe was fixed in 4% formalin for 24 hours, then embedded in paraffin. Four-μm thick sections were prepared and stained with hematoxylin and eosin (H&E) to evaluate the overall liver morphology. Sirius Red/Fast Green (SRFG) staining was used to visualize collagen deposition. Immunohistochemistry was performed to identify cholangiocytes using an anti-CK19 primary antibody (1:1000 dilution, ab52625, Abcam), followed by incubation with an appropriate peroxidase-conjugated secondary antibody. Immune complexes were visualized using 3,3’-diaminobenzidine (DAB). Digital images were acquired using a Hamamatsu NanoZoomer (Hamamatsu Photonics), and ImageJ software was used to quantify collagen deposition and CK19-positive areas, expressed as a percentage of the total image field. Digital HE slides of FFPE serial sections were analyzed by a board-certified veterinary pathologist for the semi-quantitative assessment of single-cell death, pigmented macrophages, and inflammation. Any form of liver damage can result in hepatocyte death through necrosis, apoptosis, or another form of cell death, which are not morphologically distinguishable, collectively referred to here as single-cell death. Scoring of these features was performed in 10 randomly selected high-powered fields (20×) using a scale of 0 = absent/minimal, 1 = mild, 2 = moderate, and 3 = severe. A mild degree of single-cell death constituted 0 to 2 hypereosinophilic cells with nuclear fading in each high-powered field. Moderate cell death involved 2 to 5 dead cells, and severe cell death was scored only when >5 necrotic cells were seen in each high-powered field. Pigmented macrophages are indicative of phagocytosis of lipid-iron-containing materials and are associated with cellular damage; these were scored according to Kleiner et al[74] as none/rare = 0, many = 1. Inflammation was scored semi-quantitatively where 0 = minimal, 1 = mild (for example, <1 inflammatory foci per 40× field), 2 = moderate (approximately 1–2 inflammatory foci per 40× field), and 3 = severe marked inflammatory infiltrates in the parenchyma and/or in portal areas/sites of ductular reaction.

RNA FISH Assay

To detect and visualize gene expression patterns in the mouse liver, SABER tyramide signal amplification (TSA) FISH was performed according to Ustyantsev et al.[75] Briefly, formalin-fixed paraffin-embedded (FFPE) liver sections (5 μm) were mounted onto positively charged RNase-free slides and dried overnight at 37°C. Slides were deparaffinized with xylene and ethanol and blocked with BLOXALL Endogenous Blocking Solution, Peroxidase and Alkaline Phosphatase (SP-6000-100, Vector Laboratories) to quench potential endogenous alkaline phosphatase and peroxidase activities. The slides were placed on a Sequenza staining rack for subsequent procedures. The slides were incubated with TE buffer at 70°C for 1 hour in a hybridization oven, followed by incubation with proteinase K (20 μg/mL in PBST) at 37°C for 10 minutes. After consecutive washes with PBST, WashHyb, and Prehyb, hybridization was conducted using gene-specific SABER probes (eg, *S100a4*-p27) in Hyb buffer at 50°C overnight in a hybridization oven. The sequence information of the probes is listed in Supplementary Table 1. Post-hybridization, the slides were washed with WashHyb and 2 × saline-sodium citrate containing Tween-20 (SSCTw) buffers. Secondary oligonucleotide probes complementary to the SABER concatemer sequences were hybridized at 42°C for 1 hour in

PBST containing 10% dextran sulfate. After washing, the slides were blocked with 5% horse serum and 5% Western Blocking Reagent (Roche), followed by incubation with anti-DIG/FITC-POD antibodies (Roche, 1:2000 dilution) for 45 minutes at 37°C. After subsequent washing steps, signal amplification was achieved using fluorescent tyramide (eg, CF647 [Biotium], 1:250 dilution in TSA development buffer) for 10 minutes at room temperature in the dark. Finally, the nuclei were counterstained with 4′,6-diamidino-2-phenylindole (DAPI) and mounted for imaging using VECTASHIELD Vibrance Antifade Mounting Medium (Vector Laboratories). Digital images were obtained using a Leica TCS SP8× confocal microscope.

RT-qPCR

RNA was extracted from the tissue using TRI-Reagent (Sigma-Aldrich) and converted into cDNA using Moloney Murine Leukemia Virus Reverse Transcriptase (Life Technologies) and Random Nonamers (Sigma-Aldrich). RT-qPCR was performed using a QuantStudio-3 system (Applied Biosystems) using Taqman primer-probe combinations or SYBRGreen for amplification. The expression of target genes was normalized to peptidyl-prolyl isomerase G (Ppig, Cyclophilin G) and further adjusted to the mean relative expression of the control group. The oligos used for (RT-)qPCR are listed in Supplementary Table 2.

RNA-seq Analysis

RNA-seq libraries were constructed at ERIBA Research Sequencing Facility according to the Smart-3SEQ protocol[76] using 100 ng of total RNA per sample as input. The libraries were pooled, and RNA sequencing was performed by Novogene using the NovaSeq X Plus Sequencing System with 150 bp paired-end reads. The obtained sequences were aligned to the mouse reference genome (GRCm39/mm39), and gene counts were quantified using featureCounts.[77] Counts data was regularized-logarithm transformed prior to hierarchical clustering and PCA. PCA and differential expression analyses were performed with the prcomp function and DESeq2 package (version 1.40.2)[78] in R (version 4.3.1). GSEA was based on genes ranked by the Wald statistic (stat column from DESeq2 output)[79] and was conducted using the fgsea package (version 1.26.0) in R using the hallmark gene sets (version 2023.2.Mm) from the Molecular Signatures Database,[80] whereas GSEA plots were made using ClusterProfiler (version 4.8.3), and overview of gene expression changes in the KEGG TGF-β signaling pathway was generated using the Pathview package (version 1.40.0)[81] for R. Differential expression and gene set enrichment were deemed significant when the Benjamini-Hochberg-adjusted P value was $< .05$. Kruskal-Wallis H tests followed by Conover post hoc comparisons was used to evaluate differential expression of individual genes between multiple groups (P values $< .05$ were considered statistically significant).

Targeted Proteomics

ABCB4 protein levels were quantified using targeted proteomics, following established protocols.[82,83] The isotopically labeled peptide LATDAAQVQGATGTR was used as a reference standard for ABCB4.

Lipidomics

The biliary lipidome was analyzed as previously described.[84] Briefly, internal standards were added, and lipids were extracted from 5 μL bile sample using MTBE/methanol extraction.[85] The resulting lipid extracts were concentrated and reconstituted in a running buffer composed of 10 mM ammonium acetate and dichloromethane (50:50, v/v) in methanol. Lipid analysis was performed using a flow injection analysis-tandem mass spectrometry (FIA-MS/MS) method, utilizing an ultra-high pressure liquid chromatography system (Nexera X2, Shimadzu) coupled with a QTRAP5500 mass spectrometer (SCIEX) operating in multiple-reaction-monitoring mode and equipped with a differential mobility spectrometry (DMS) interface utilizing SelexION technology. Raw data were processed, and lipid quantification was performed using the Shotgun Lipidomics Assistant, a Python-based application.[84] Lipidomics data were analyzed using the MetaboAnalyst 6.0 web tool.[86]

Enrichment Analysis of Hepatic Proteomics Data From Patients With PFIC3

Significant differentially expressed proteins (t-test adj. $P < .05$) between patients with PFIC3 and control livers were obtained from a published dataset.[64] The Sangerbox platform (http://sangerbox.com/home.html)[87] was used to conduct enrichment analysis using human-ortholog hallmark gene sets (version 7.4) from the Molecular Signatures Database.[88] Differential gene set enrichment was deemed significant when the Benjamini-Hochberg-adjusted P value was $< .05$.

Statistical Analysis

Kruskal-Wallis H tests followed by Conover post hoc comparisons were used to evaluate the significance of differences between multiple groups using Brightstat.[89] GraphPad Prism (GraphPad Software, version 8) was used to generate the graphs. P values $< .05$ were considered statistically significant.

Supplementary Material

Note: To access the supplementary material accompanying this article, visit the full text version at https://doi.org/10.1016/j.jcmgh.2026.101783.

References

1. Morotti RA, Suchy FJ, Magid MS. Progressive familial intrahepatic cholestasis (PFIC) type 1, 2, and 3: a review of the liver pathology findings. Semin Liver Dis 2011; 31:3–10.

2. Jacquemin E. Progressive familial intrahepatic cholestasis. Clin Res Hepatol Gastroenterol 2012; 36:S26–S35.
3. Deleuze JF, Jacquemin E, Dubuisson C, et al. Defect of multidrug-resistance 3 gene expression in a subtype of progressive familial intrahepatic cholestasis. Hepatology 1996;23:904–908.
4. de Vree JM, Jacquemin E, Sturm E, et al. Mutations in the MDR3 gene cause progressive familial intrahepatic cholestasis. Proc Natl Acad Sci U S A 1998;95:282–287.
5. Smit JJ, Schinkel AH, Oude Elferink RP, et al. Homozygous disruption of the murine mdr2 P-glycoprotein gene leads to a complete absence of phospholipid from bile and to liver disease. Cell 1993;75:451–462.
6. Smith AJ, Timmermans-Hereijgers JL, Roelofsen B, et al. The human MDR3 P-glycoprotein promotes translocation of phosphatidylcholine through the plasma membrane of fibroblasts from transgenic mice. FEBS Lett 1994;354:263–266.
7. Groen A, Romero MR, Kunne C, et al. Complementary functions of the flippase ATP8B1 and the floppase ABCB4 in maintaining canalicular membrane integrity. Gastroenterology 2011;141:1927–1937.e1–4.
8. Colombo C, Vajro P, Degiorgio D, et al, SIGENP Study Group for Genetic Cholestasis. Clinical features and genotype-phenotype correlations in children with progressive familial intrahepatic cholestasis type 3 related to ABCB4 mutations. J Pediatr Gastroenterol Nutr 2011; 52:73–83.
9. Jacquemin E, De Vree JM, Cresteil D, et al. The wide spectrum of multidrug resistance 3 deficiency: from neonatal cholestasis to cirrhosis of adulthood. Gastroenterology 2001;120:1448–1458.
10. Davit-Spraul A, Fabre M, Branchereau S, et al. ATP8B1 and ABCB11 analysis in 62 children with normal gamma-glutamyl transferase progressive familial intrahepatic cholestasis (PFIC): phenotypic differences between PFIC1 and PFIC2 and natural history. Hepatology 2010;51:1645–1655.
11. Khabou B, Mahjoub B, Barbu V, et al. Phenotypic variability in Tunisian PFIC3 patients harboring a complex genotype with a differential clinical outcome of UDCA treatment. Clin Chim Acta 2018;486:122–128.
12. Sutton H, Sokol RJ, Kamath BM. IBAT inhibitors in pediatric cholestatic liver diseases: transformation on the horizon? Hepatology 2024;82:985–995.
13. Deeks ED. Odevixibat: first approval. Drugs 2021; 81:1781–1786.
14. Aronson SJ, Bakker RS, Shi X, et al. Liver-directed gene therapy results in long-term correction of progressive familial intrahepatic cholestasis type 3 in mice. J Hepatol 2019;71:153–162.
15. Wei G, Cao J, Huang P, et al. Synthetic human ABCB4 mRNA therapy rescues severe liver disease phenotype in a BALB/c.Abcb4 mouse model of PFIC3. J Hepatol 2021;74:1416–1428.
16. Weber ND, Odriozola L, Martínez-García J, et al. Gene therapy for progressive familial intrahepatic cholestasis type 3 in a clinically relevant mouse model. Nat Commun 2019;10:5694.
17. Jones-Hughes T, Campbell J, Crathorne L. Epidemiology and burden of progressive familial intrahepatic cholestasis: a systematic review. Orphanet J Rare Dis 2021;16:255.
18. Mehl A, Bohorquez H, Serrano MS, et al. Liver transplantation and the management of progressive familial intrahepatic cholestasis in children. World J Transplant 2016;6:278–290.
19. Ikenaga N, Liu SB, Sverdlov DY, et al. A new Mdr2(-/-) mouse model of sclerosing cholangitis with rapid fibrosis progression, early-onset portal hypertension, and liver cancer. Am J Pathol 2015;185:325–334.
20. Behary J, Raposo AE, Amorim NML, et al. Defining the temporal evolution of gut dysbiosis and inflammatory responses leading to hepatocellular carcinoma in Mdr2 -/- mouse model. BMC Microbiol 2021;21:113.
21. Lamireau T, Bouchard G, Yousef IM, et al. Dietary lecithin protects against cholestatic liver disease in cholic acid-fed Abcb4- deficient mice. Pediatr Res 2007; 61:185–190.
22. Perwaiz S, Forrest D, Mignault D, et al. Appearance of atypical 3 alpha,6 beta,7 beta,12 alpha-tetrahydroxy-5 beta-cholan-24-oic acid in spgp knockout mice. J Lipid Res 2003;44:494–502.
23. de Boer JF, Verkade E, Mulder NL, et al. A human-like bile acid pool induced by deletion of hepatic Cyp2c70 modulates effects of FXR activation in mice. J Lipid Res 2020;61:291–305.
24. Takahashi S, Fukami T, Masuo Y, et al. Cyp2c70 is responsible for the species difference in bile acid metabolism between mice and humans. J Lipid Res 2016;57:2130–2137.
25. de Boer JF, de Vries HD, Palmiotti A, et al. Cholangiopathy and biliary fibrosis in Cyp2c70-deficient mice are fully reversed by ursodeoxycholic acid. Cell Mol Gastroenterol Hepatol 2021;11:1045–1069.
26. Honda A, Miyazaki T, Iwamoto J, et al. Regulation of bile acid metabolism in mouse models with hydrophobic bile acid composition. J Lipid Res 2020;61:54–69.
27. Miethke AG, Moukarzel A, Porta G, et al. Maralixibat in progressive familial intrahepatic cholestasis (MARCH-PFIC): a multicentre, randomised, double-blind, placebo-controlled, phase 3 trial. Lancet Gastroenterol Hepatol 2024;9:620–631.
28. Ovchinsky N, Aumar M, Baker A, et al. Efficacy and safety of odevixibat in patients with Alagille syndrome (ASSERT): a phase 3, double-blind, randomised, placebo-controlled trial. Lancet Gastroenterol Hepatol 2024;9:632–645.
29. Fuchs CD, Paumgartner G, Mlitz V, et al. Colesevelam attenuates cholestatic liver and bile duct injury in Mdr2-/- mice by modulating composition, signalling and excretion of faecal bile acids. Gut 2018;67:1683–1691.
30. Palmiotti A, de Vries HD, Hovingh MV, et al. Bile acid sequestration via colesevelam reduces bile acid hydrophobicity and improves liver pathology in Cyp2c70-/- mice with a human-like bile acid composition. Biomedicines 2023;11:2495.
31. Verkade HJ, Havinga R, Shields DJ, et al. The phosphatidylethanolamine N-methyltransferase pathway is

quantitatively not essential for biliary phosphatidylcholine secretion. J Lipid Res 2007;48:2058–2064.
32. Haal S, Guman MSS, Acherman YIZ, et al. Gallstone formation follows a different trajectory in bariatric patients compared to nonbariatric patients. Metabolites 2021;11:682.
33. Suzuki N, Irie M, Iwata K, et al. Altered expression of alkaline phosphatase (ALP) in the liver of primary biliary cirrhosis (PBC) patients. Hepatol Res 2006;35:37–44.
34. Penn DJ, Zala SM, Luzynski KC. Regulation of sexually dimorphic expression of major urinary proteins. Front Physiol 2022;13:822073.
35. Gao R, Wang H, Li T, et al. Secreted MUP1 that reduced under ER stress attenuates ER stress induced insulin resistance through suppressing protein synthesis in hepatocytes. Pharmacol Res 2023;187:106585.
36. Chen Y, Fan Y, Guo D-Y, et al. Study on the relationship between hepatic fibrosis and epithelial-mesenchymal transition in intrahepatic cells. Biomed Pharmacother 2020;129:110413.
37. Sterzer V, Alsamman M, Bretag T, et al. EMT in liver fibrosis. Curr Pathobiol Rep 2014;2:201–207.
38. Fendt BM, Hirschmann A, Bruns M, et al. Protein atlas of fibroblast specific protein 1 (FSP1)/S100A4. Histol Histopathol 2023;38:1391–1401.
39. Kalluri R, Neilson EG. Epithelial-mesenchymal transition and its implications for fibrosis. J Clin Invest 2003; 112:1776–1784.
40. Baghdasaryan A, Fuchs CD, Österreicher CH, et al. Inhibition of intestinal bile acid absorption improves cholestatic liver and bile duct injury in a mouse model of sclerosing cholangitis. J Hepatol 2016;64:674–681.
41. Truong JK, Bennett AL, Klindt C, et al. Ileal bile acid transporter inhibition in Cyp2c70 KO mice ameliorates cholestatic liver injury. J Lipid Res 2022;63:100261.
42. Voshol PJ, Minich DM, Havinga R, et al. Postprandial chylomicron formation and fat absorption in multidrug resistance gene 2 P-glycoprotein-deficient mice. Gastroenterology 2000;118:173–182.
43. Hulzebos CV, Voshol PJ, Wolters H, et al. Bile duct proliferation associated with bile salt-induced hypercholeresis in Mdr2 P-glycoprotein-deficient mice. Liver Int 2005;25:604–612.
44. Van Nieuwkerk CM, Elferink RP, Groen AK, et al. Effects of ursodeoxycholate and cholate feeding on liver disease in FVB mice with a disrupted mdr2 P-glycoprotein gene. Gastroenterology 1996;111:165–171.
45. Madry C, Elbahnsi A, Delaunay J-L, et al. ABCB4 disease-causing variants S242R, S346I, T437I and T1077M significantly impair its function and display differential sensitivity to potentiators. Sci Rep 2025;15:44544.
46. Bell EL, Truong JK, Jo Y, et al. An ABCB11 variant registry and novel knockin mouse model of PFIC2 based on the clinically relevant ABCB11 E297G variant. J Lipid Res 2025; 66:100840.
47. Pawlikowska L, Groen A, Eppens EF, et al. A mouse genetic model for familial cholestasis caused by ATP8B1 mutations reveals perturbed bile salt homeostasis but no impairment in bile secretion. Hum Mol Genet 2004;13:881–892.
48. Wang R, Chen HL, Liu L, et al. Compensatory role of P-glycoproteins in knockout mice lacking the bile salt export pump. Hepatology 2009;50:948–956.
49. Kok T, Hulzebos CV, Wolters H, et al. Enterohepatic circulation of bile salts in farnesoid X receptor-deficient mice: efficient intestinal bile salt absorption in the absence of ileal bile acid-binding protein. J Biol Chem 2003;278:41930–41937.
50. Verkade HJ, Vonk RJ, Kuipers F. New insights into the mechanism of bile acid-induced biliary lipid secretion. Hepatology 1995;21:1174–1189.
51. Gurantz D, Hofmann AF. Influence of bile acid structure on bile flow and biliary lipid secretion in the hamster. Am J Physiol 1984;247:G736–G748.
52. Wang R, Sheps JA, Liu L, et al. Hydrophilic bile acids prevent liver damage caused by lack of biliary phospholipid in Mdr2-/- mice. J Lipid Res 2019;60:85–97.
53. Stephenson DJ, MacKnight HP, Hoeferlin LA, et al. A rapid and adaptable lipidomics method for quantitative UPLC-mass spectrometric analysis of phosphatidylethanolamine and phosphatidylcholine in vitro, and in cells. Anal Methods 2019;11:1765–1776.
54. Su KC, Wu YC, Chen CS, et al. Bile acids increase alveolar epithelial permeability via mitogen-activated protein kinase, cytosolic phospholipase A2 , cyclooxygenase-2, prostaglandin E2 and junctional proteins. Respirology 2013; 18:848–856.
55. Han MS, Park SY, Shinzawa K, et al. Lysophosphatidylcholine as a death effector in the lipoapoptosis of hepatocytes. J Lipid Res 2008;49:84–97.
56. Liu P, Zhu W, Chen C, et al. The mechanisms of lysophosphatidylcholine in the development of diseases. Life Sci 2020;247:117443.
57. Kakisaka K, Cazanave SC, Fingas CD, et al. Mechanisms of lysophosphatidylcholine-induced hepatocyte lipoapoptosis. Am J Physiol Gastrointest Liver Physiol 2012;302:G77–G84.
58. Coleman JA, Quazi F, Molday RS. Mammalian P4-ATPases and ABC transporters and their role in phospholipid transport. Biochim Biophys Acta 2013;1831:555–574.
59. Morita S, Tsuda T, Horikami M, et al. Bile salt-stimulated phospholipid efflux mediated by ABCB4 localized in nonraft membranes. J Lipid Res 2013;54:1221–1230.
60. Asamoto Y, Tazuma S, Ochi H, et al. Bile-salt hydrophobicity is a key factor regulating rat liver plasma-membrane communication: relation to bilayer structure, fluidity and transporter expression and function. Biochem J 2001;359:605–610.
61. Yousef IM, Barnwell S, Gratton F, et al. Liver cell membrane solubilization may control maximum secretory rate of cholic acid in the rat. Am J Physiol 1987; 252:G84–G91.
62. Heldin C-H, Landström M, Moustakas A. Mechanism of TGF-beta signaling to growth arrest, apoptosis, and epithelial-mesenchymal transition. Curr Opin Cell Biol 2009;21:166–176.
63. Rygiel KA, Robertson H, Marshall HL, et al. Epithelial-mesenchymal transition contributes to portal tract fibrogenesis during human chronic liver disease. Lab Invest 2008;88:112–123.

64. Guerrero L, Carmona-Rodríguez L, Santos FM, et al. Molecular basis of progressive familial intrahepatic cholestasis 3. A proteomics study. Biofactors 2024; 50:794–809.
65. Munker S, Wu Y-L, Ding H-G, et al. Can a fibrotic liver afford epithelial-mesenchymal transition? World J Gastroenterol 2017;23:4661–4668.
66. Tsuruya K, Yokoyama K, Mishima Y, et al. Abcb4-defect cholangitis mouse model with hydrophobic bile acid composition by in vivo liver-specific gene deletion. J Lipid Res 2024;65:100616.
67. Klindt C, Truong JK, Bennett AL, et al. Hepatic bile acid accretion correlates with cholestatic liver injury and therapeutic response in Cyp2c70 knockout mice with a humanized bile acid composition. Am J Physiol Gastrointest Liver Physiol 2024;327:G789–G809.
68. Ghallab A, Kunz S, Drossel C, et al. Validation of NBD-coupled taurocholic acid for intravital analysis of bile acid transport in liver and kidney of mice. EXCLI J 2024; 23:1330–1352.
69. Fedoseienko A, Wijers M, Wolters JC, et al. The COMMD family regulates plasma LDL levels and attenuates atherosclerosis through stabilizing the CCC complex in endosomal LDLR trafficking. Circ Res 2018; 122:1648–1660.
70. Verkade E, Shen W, Hovingh MV, et al. Gut microbiota depletion aggravates bile acid-induced liver pathology in mice with a human-like bile acid composition. Clin Sci (Lond) 2023;137:1637–1650.
71. Heuman DM. Quantitative estimation of the hydrophilic-hydrophobic balance of mixed bile salt solutions. J Lipid Res 1989;30:719–730.
72. Böttcher CJF, van Gent CM, Pries CN. A rapid and sensitive sub-micro phosphorus determination. Anal Chim Acta 1961;24:203–204.
73. Bligh EG, Dyer WJ. A rapid method of total lipid extraction and purification. Can J Biochem Physiol 1959;37:911–917.
74. Kleiner DE, Brunt EM, Van Natta M, et al, Nonalcoholic Steatohepatitis Clinical Research Network. Design and validation of a histological scoring system for nonalcoholic fatty liver disease. Hepatology 2005;41:1313–1321.
75. Ustyantsev K, Stranges M, Volpe FG, et al. One probe fits all: a highly customizable modular RNA in situ hybridization platform expanding the application of SABER DNA probes. Development 2025;152:dev204775.
76. Foley JW, Zhu C, Jolivet P, et al. Gene expression profiling of single cells from archival tissue with laser-capture microdissection and Smart-3SEQ. Genome Res 2019;29:1816–1825.
77. Liao Y, Smyth GK, Shi W. featureCounts: an efficient general purpose program for assigning sequence reads to genomic features. Bioinformatics 2014;30:923–930.
78. Love MI, Huber W, Anders S. Moderated estimation of fold change and dispersion for RNA-seq data with DESeq2. Genome Biol 2014;15:550.
79. Subramanian A, Tamayo P, Mootha VK, et al. Gene set enrichment analysis: a knowledge-based approach for interpreting genome-wide expression profiles. Proc Natl Acad Sci U S A 2005;102:15545–15550.
80. Castanza AS, Recla JM, Eby D, et al. Extending support for mouse data in the Molecular Signatures Database (MSigDB). Nat Methods 2023;20:1619–1620.
81. Luo W, Brouwer C. Pathview: an R/Bioconductor package for pathway-based data integration and visualization. Bioinformatics 2013;29:1830–1831.
82. Wolters JC, Ciapaite J, van Eunen K, et al. Translational targeted proteomics profiling of mitochondrial energy metabolic pathways in mouse and human samples. J Proteome Res 2016;15:3204–3213.
83. Schonewille M, de Boer JF, Mele L, et al. Statins increase hepatic cholesterol synthesis and stimulate fecal cholesterol elimination in mice. J Lipid Res 2016; 57:1455–1464.
84. Su B, Bettcher LF, Hsieh WY, et al. A DMS shotgun lipidomics workflow application to facilitate high-throughput, comprehensive lipidomics. J Am Soc Mass Spectrom 2021;32:2655–2663.
85. Matyash V, Liebisch G, Kurzchalia TV, et al. Lipid extraction by methyl-tert-butyl ether for high-throughput lipidomics. J Lipid Res 2008;49:1137–1146.
86. Pang Z, Lu Y, Zhou G, et al. MetaboAnalyst 6.0: towards a unified platform for metabolomics data processing, analysis and interpretation. Nucleic Acids Res 2024; 52:W398–W406.
87. Chen D, Xu L, Xing H, et al. Sangerbox 2: enhanced functionalities and update for a comprehensive clinical bioinformatics data analysis platform. Imeta 2024;3:e238.
88. Liberzon A, Subramanian A, Pinchback R, et al. Molecular signatures database (MSigDB) 3.0. Bioinformatics 2011;27:1739–1740.
89. Stricker D. BrightStat.com: free statistics online. Comput Methods Programs Biomed 2008;92:135–143.

Received October 17, 2025. Accepted March 31, 2026.

Correspondence
Address correspondence to: Folkert Kuipers, PhD, European Research Institute for the Biology of Ageing (ERIBA), University Medical Center Groningen, Hanzeplein 1, Groningen 9713 GZ, The Netherlands. e-mail: f.kuipers@umcg.nl; or Jan Freark de Boer, PhD, Department of Pediatrics, University Medical Center Groningen, Hanzeplein 1, Groningen 9713 GZ, The Netherlands. e-mail: j.f.de.boer@umcg.nl.

Acknowledgments
The authors thank Mariska de Graaf (Department of Pediatrics and Interfaculty Mass Spectrometry Center, University Medical Center, University of Groningen) for expert technical assistance.

CRediT Authorship Contributions

Lixin Ke (Investigation: Lead; Methodology: Lead; Project administration: Equal; Writing – original draft: Lead)

Niels L. Mulder (Formal analysis: Equal; Methodology: Equal; Writing – review & editing: Supporting)

Milaine V. Hovingh (Formal analysis: Equal; Methodology: Equal; Writing – review & editing: Supporting)

Rick Havinga (Investigation: Equal; Methodology: Supporting; Writing – review & editing: Supporting)

Ellen Weersing (Investigation: Supporting; Methodology: Supporting; Writing – review & editing: Supporting)
Hilde D. de Vries (Formal analysis: Equal; Investigation: Supporting; Writing – review & editing: Supporting)
Krisztina de Bruyn (Formal analysis: Equal; Methodology: Equal; Writing – review & editing: Supporting)
Kirill Ustyantsev (Investigation: Equal; Methodology: Equal; Writing – review & editing: Supporting)
Eugene Berezikov (Investigation: Equal; Methodology: Equal; Writing – review & editing: Supporting)
Vincent W. Bloks (Methodology: Supporting; Validation: Supporting; Writing – review & editing: Supporting)
Brecht Attema (Formal analysis: Equal; Methodology: Equal; Writing – review & editing: Supporting)
Anna Worthmann (Formal analysis: Equal; Methodology: Equal; Writing – review & editing: Supporting)
Joerg Heeren (Formal analysis: Supporting; Investigation: Supporting; Resources: Equal; Writing – review & editing: Supporting)
Rachel Tiessen-Thomas, PhD (Formal analysis: Lead)
Justina C. Wolters (Data curation: Lead; Formal analysis: Equal; Writing – review & editing: Supporting)
Henkjan J. Verkade (Conceptualization: Equal; Supervision: Equal; Writing – review & editing: Equal)
Folkert Kuipers, PhD (Conceptualization: Equal; Funding acquisition: Lead; Supervision: Equal; Writing – review & editing: Equal)
Jan Freark de Boer (Conceptualization: Lead; Formal analysis: Equal; Funding acquisition: Equal; Resources: Equal; Supervision: Equal; Writing – review & editing: Lead)

Conflicts of interest

The authors disclose no conflicts.

Funding

Lixin Ke is supported by the China Scholarship Council (CSC No. 202206380051) and funded by De-Cock-Hadders Foundation (O/817446). Henkjan J. Verkade has received unrestricted research grants from Albireo AB/Ipsen and Mirum. Folkert Kuipers is supported by unrestricted grant from the Noaber Foundation, Lunteren, The Netherlands. Jan Freark de Boer is supported by an NWO-Demonstrator grant (2024/TTW/01761283). The funders had no role in the study design, data collection and analyses, interpretation of data, decision to publish, or preparation of the manuscript.

Data Availability

All data are available upon reasonable request.

Cellular and Molecular Gastroenterology and Hepatology, Volume *20*

Supplemental information

Bile Acid Detergency as Determinant of Liver Pathology in a Humanized Mouse Model of Progressive Familial Intrahepatic Cholestasis Type 3

Lixin Ke, Niels L. Mulder, Milaine V. Hovingh, Rick Havinga, Ellen Weersing, Hilde D. de Vries, Krisztina de Bruyn, Kirill Ustyantsev, Eugene Berezikov, Vincent W. Bloks, Brecht Attema, Anna Worthmann, Joerg Heeren, Justina C. Wolters, Rachel Tiessen-Thomas, Henkjan J. Verkade, Folkert Kuipers, and Jan Freark de Boer

SUPPLEMENTAL MATERIAL

Supplemental Table S1. Primary probe sequences used in RNA fluorescence in situ hybridization (FISH) assay.

Gene	Probe_ID	Sequence 5’-3’ with p27 3’ initiator sequence (tttCATCATCAT)
m*S100a4*	1	AACCTTTTATTGAATTTGCTCAGCACTGTGCACATGTGCtttCATCATCAT
	2	AGTAAGGCACTATGCTCACAGCCAACAGGGAAGATCCtttCATCATCAT
	3	TGGCAAACTACACCCCAACACTTCATCTGAGGAGTCTtttCATCATCAT
	4	CCTTATCTGGGCAGCCCTCAAAGAATTCATTGCACATtttCATCATCAT
	5	CAATGCAGGACAGGAAGACACAGTACTCCTGGAAGTCtttCATCATCAT
	6	CATTGTCCCTGTTGCTGTCCAAGTTGCTCATCACCTTtttCATCATCAT
	7	CCCTGGTCAGTAGCTCCTTGAGCTCTGTCTTGTTCAGtttCATCATCAT
	8	ACTTGTCACCCTCTTTGCCTGAGTATTTGTGGAAGGTtttCATCATCAT
	9	TTGCCATGGTAACCGTTGAGACCAGACCAAGAGAGAGtttCATCATCAT

Supplemental Table S2. Taqman and SYBR Green PCR primer and probe sequences.

Gene		Taqmanoligo's (5'→3')
Col1a1	Fw	CGG CTC CTG CTC CTC TTA GG
	Rv	CTG ACT TCA GGG ATG TCT TCT TGG
	Probe	CCA CTG CCC TCC TGA CGC ATG G
Mmp12	Fw	ATC TTA GAG CAG TGC CCC AG
	Rv	CAT CCT CAC GCT TCA TGT CC
	Probe	TCA AGA TGG ATG AAG CGG TAC CTC AC
Timp1	Fw	TCT GAG CCC TGC TCA GCA A
	Rv	AAC AGG GAA ACA CTG TGC ACA C
	Probe	CCA CAG CCA GCA CTA TAG GTC TTT GAG AAA GC
Tnf	Fw	GTA GCC CAC GTC GTA GCA AAC
	Rv	AGT TGG TTG TCT TTG AGA TCC ATG
	Probe	CGC TGG CTC AGC CAC TCC AGC
Krt19	Fw	CAC CTA CCT TGC TCG GAT TGA
	Rv	CCG TGA CTT CGG TCT TGC TT
	Probe	ACA CCC AGG TCG CCG TCC ACT
Ppig	Fw	CAG ATC GAG GGA TCG ATT CAG
	Rv	TCA CCA CTT GAC ACC CTC ATT C
	Probe	CTC CTC CAC ATT GGA GAC AAG AGA TGC A

Gene		SYBR Green oligo's (5'→3')
Pemt	Fw	AGG AGT CCA GAG TGA CCA CAT TTC
	Rv	AGG AGA GCA ACC ACG TAG AC
Pcyt1a	Fw	CTG CAG TGA TGA GCT AAC GC
	Rv	GGC GAC AAA ATC AAT CCG GTG
Ppig	Fw	GGG GAT AAA GGT CCA GCG T
	Rv	AGA CAA CTC TTC CAG CAG GT
Pla2g4a	Fw	CAG CAC ATT ATA GTG GAA CAC CA
	Rv	AGT GTC CAG CAT ATC GCC AAA
Lpcat3	Fw	GGC CTC TCA ATT GCT TAT TT
	Rv	AGG ATG AGG AAC TGA AGA AC
